# Acute Loss of Tactile Input Leads to General Compensatory Changes in Eye–Hand Coordination during Object Manipulation

**DOI:** 10.1523/ENEURO.0487-23.2025

**Published:** 2025-09-25

**Authors:** Kevin Ung, John F. Magnotti, Brandon Kim, Jeffrey M. Yau, Per F. Nordmark

**Affiliations:** ^1^Department of Neuroscience, Baylor College of Medicine, Houston, Texas 77030; ^2^Department of Neurosurgery, Perelman School of Medicine, University of Pennsylvania, Philadelphia, Pennsylvania 19104; ^3^Department of Medical and Translational Biology, and Department of Diagnostics and Intervention, Section for Hand and Plastic Surgery, Umeå University, Umeå 901 87, Sweden

**Keywords:** digital anesthesia, eye-hand coordination, manual dexterity, sensorimotor integration, somatosensory feedback, visual compensation

## Abstract

Current models of motor control emphasize the critical role of sensory feedback, as demonstrated by movement coordination deficits following sensory impairment. When both vision and touch are available for object-oriented manual behaviors, they serve distinct roles; vision guides the execution of planned movements, while touch provides more direct feedback on hand–object interactions. The impact of losing somatosensory feedback on eye–hand coordination during dexterous object manipulation tasks has not been thoroughly studied. Conceivably, vision is recruited to compensate for the feedback lost when touch is abolished based on the dexterity demands of the behavior. To investigate this, we tested healthy participants of either sex on a manual dexterity task requiring the movement of small metal pegs, both before and after the administration of digital anesthesia, which selectively abolished cutaneous sensations in the fingertips while preserving motor function. We recorded participants' gaze and hand positions. Despite loss of cutaneous feedback, participants successfully completed the pegboard task. However, they exhibited significantly longer trial times and altered force profiles. Notably, acute somatosensory loss triggered a rapid shift in visual behavior, characterized by a tighter coupling between gaze and hand positions across all task actions, even those not directly involving object manipulation. These changes, which occurred with anesthesia of the dominant and nondominant hands, were not evident with sham (saline) injections. Our findings underscore the contributions of sensory feedback to force control in service of dexterous object manipulation and reveal the nonselective nature of compensatory gaze–hand coordination processes.

## Significance Statement

Touch and vision typically support distinct but coordinated aspects of dexterous manual behaviors. Here, we evaluated how acute removal of tactile feedback using digital anesthesia affected performance and gaze–hand coordination in a manual dexterity task. With insensate fingers and intact vision, participants continued to perform the task successfully, albeit with marked longer trial times and altered force profiles. We also observed closer alignment between gaze and hand positions during all task actions, even those that did not involve object manipulation. Our results reveal the consequences of acute somatosensory loss and the general nature of compensatory eye–hand coordination processes.

## Introduction

Dexterous manual interactions with objects require coordination between sensory and motor systems. When all sensory channels are available, vision and touch support complementary roles: vision provides information that supports grasping and movement planning (Johansson et al., 2001), while touch provides direct feedback for performance monitoring and error correction (Johansson and Flanagan, 2009). When tactile sensory inputs are diminished or lost, the available input channels from vision may be recommissioned to fill in critical information gaps.

Touch is crucial for perceiving hand posture, movements, and the physical interactions between the hand and grasped objects (Johansson and Flanagan, 2009). Tactile signals from proprioceptors and cutaneous mechanoreceptors inform grasped objects' properties, including shape (Johnson and Hsiao, 1992; Jenmalm et al., 2003), grip force adaptation (Jenmalm et al., 2000), alignment of fingertips (Monzée et al., 2003), and texture or friction (Johansson and Westling, 1984; Sathian et al., 1989; Bilaloglu et al., 2016). Additionally, touch provides real-time feedback about forces (Johansson and Westling, 1987), friction, and slip events during object manipulation (Johansson and Westling, 1987; Birznieks et al., 1998). The absence or reduction of tactile feedback disrupts these critical functions, leading to impaired manual dexterity and object manipulation deficits.

Vision, while less directly involved in signaling mechanical interactions, supports manual tasks by providing information critical for object localization (Johansson et al., 2001), shape inference, and contextual cues for prehension (Gordon et al., 1993; Jenmalm and Johansson, 1997; Jenmalm et al., 2000; Winges et al., 2003; Lukos et al., 2007; Johansson and Flanagan, 2009). Gaze direction is integral to planning and monitoring visually guided actions (Johansson et al., 2001; Sailer et al., 2005). Individuals who have suffered nerve injuries affecting fingertip sensation often express a greater reliance on visual feedback when engaging in dexterous object manipulation tasks (Blouin et al., 1996; Vercher et al., 1996; Stenneken et al., 2006). Nerve injury is also associated with increased gray matter volume in regions of visual association cortex linked to eye–hand coordination, consistent with an increased dependence on vision to compensate for reduced somatosensory input (Nordmark et al., 2018). Such compensatory changes may be considered a form of sensorimotor learning, where the brain adapts to sensory deficits by modifying feedback processing.

Effective sensorimotor behavior requires the association of actions and their sensory consequences. Severe deafferentation such as for an entire limb or the whole body is known to cause changes in gross motor function, timing of movements, and prehension (Gentilucci et al., 1994, 1997; Blouin et al., 1996; Cuadra et al., 2019). Individuals who have lost their somatosensory and proprioceptive feedback develop movement disorders (Rao and Gordon, 2001; Chesler et al., 2016; Mahmud et al., 2017; Yahya et al., 2019) and ultimately compensate for the loss of input through other mechanisms (Jenmalm and Johansson, 1997; Augurelle et al., 2003; Monzée et al., 2003; Trillos et al., 2018). However, basic motor tasks such as lifting an object, holding it, and lowering it to the surface may still be performed well as sufficient force to maintain grasping the object can be produced simply by applying more force (Jenmalm et al., 2000). Motor movements can sometimes be informed by the context in which the movement occurred, based on the goal and the sensory information available (Sober and Sabes, 2005). With hand–object interactions, visual processing that typically subserves reaching and grasping may be recruited to provide feedback to compensate for missing or impoverished somatosensory signaling. Whether this compensation­ is restricted to specific task states (i.e., evaluating whether an object is successfully grasped) or generalizes across contexts is unknown.

In two experiments, we asked how the loss of tactile feedback in the fingers affects gaze behavior during a fine motor task testing manual dexterity and whether compensatory gaze changes are specific or generalized. To address these questions, we tracked hand movements and gaze direction as participants performed a pegboard test that required the collection, transport, and placement of small pegs. We assessed performance before and after we abolished tactile feedback through local anesthesia of the digits. We hypothesized that the loss of somatosensory feedback would induce compensatory gaze changes that were specific to only action contexts involving precise object manipulation. We found that removal of tactile feedback perturbed performance which visual compensation only partially rescued. Notably, compensatory gaze changes were not restricted to specific actions in the pegboard task and instead reflected a general yoking of gaze to the anesthetized hand. These behavioral changes, which were minimal with saline injections, occurred with anesthesia of the nondominant hand (Experiment 1) and dominant hand (Experiment 2).

## Materials and Methods

### Participants

Eleven healthy individuals participated in the study (mean age, 29 years; range, 23–42 years; four females). Ten participants were right-hand dominant (Edinburgh inventory, laterality index 70–100; Caplan and Mendoza, 2011) and one left-hand dominant (−80). Eight of the participants were right-eye dominant, two left-eye dominant, and one bilaterally dominant. All participants had normal or corrected-to-normal vision.

Five participants performed the task (Experiment 1) under baseline (no sham or anesthesia) conditions and after digital anesthesia to the nondominant hand. A separate sample of six participants performed the identical task (Experiment 2) under baseline conditions, saline injection to the dominant hand (sham), and digital anesthesia to the dominant hand. Beyond serving as a replication study, Experiment 2 provided the opportunity to control for potential hand dominance. Data related to gaze position for one participant in Experiment 2 were dropped from analyses due to a technical problem with gaze tracking.

All participants gave their written informed consent in accordance with the Declaration of Helsinki, and the Swedish Ethics Review Authority approved the study (Dnr 2021-06981-01). No participants reported a history of nerve disorders or diabetes. All participants were paid for their participation (500 SEK).

### General overview

**Figure 1.**
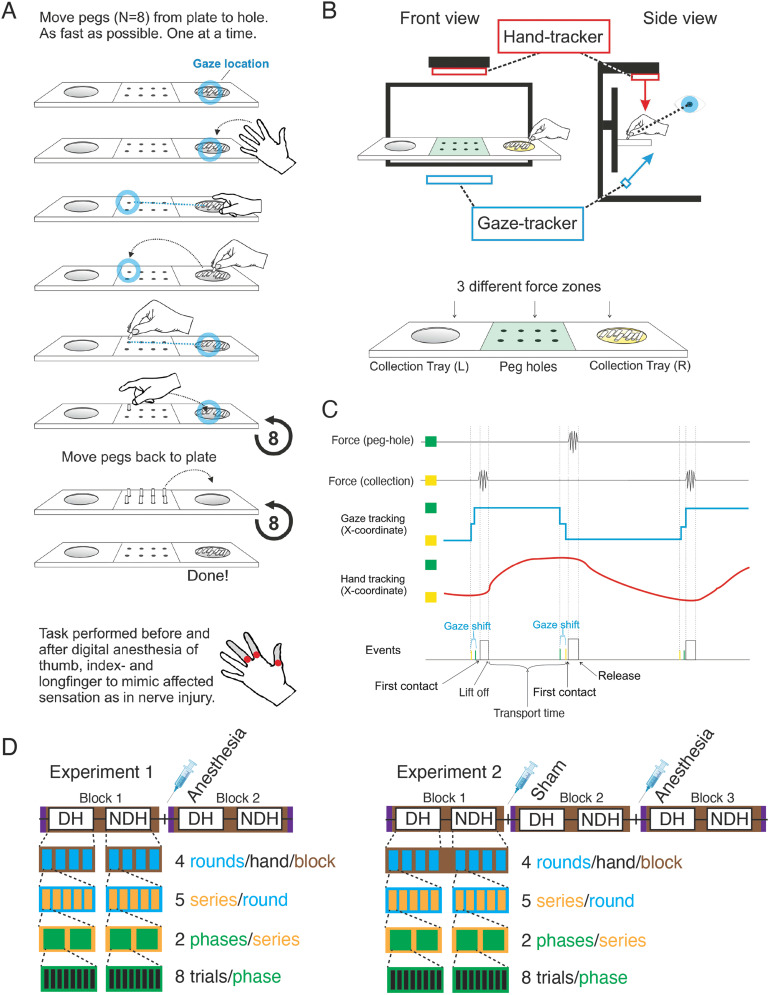
Overview of the workspace, task, and recorded data. ***A***, Schematic of the manual dexterity task. Participants collect an individual peg from the collection tray and deliver the pegs to isolated holes in the delivery tray. This action is performed sequentially for all eight pegs. Each tray has force transducers collecting normal and torque forces. ***B***, Layout of the positioning for the hand tracker, gaze tracker, and pegboard. ***C***, Example traces of the force tracking, hand tracking, and eye tracking during the task. ***D***, Schematic depicting the task structure in Experiment 1 (left) and Experiment 2 (right). Each white box represents a hand within a block. Black ticks represent injection timepoints. The purple box represents 2PD tests. Within each block, there are four rounds (teal) of data collection where each round contains five series (orange) with two phases (green). The placement phase comprised eight pegs (black) collected and delivered sequentially followed by the retrieval phase, totaling 160 peg placements and 160 peg retrievals per hand per block. DH, dominant hand; NDH, nondominant hand.

The participants performed a dexterous unimanual task comprising the collection, transport, and delivery of pegs (Fig. 1*A*). A fixed forehead support defined the individual location of the head and eyes during performance of the task. Gaze and hand positions in the workspace were recorded throughout the performance of the task using recording devices mounted in fixed positions on a height-adjustable desk (Fig. 1*B*). A monitor (480 × 270 mm) defined the depth of the workspace and displayed written instructions to the participant during the tasks. A 90 mm deep shelf with collection (outer) trays and the pegboard (middle) tray was mounted 165 mm from the top of the computer screen, serving as the basis for the workspace. Force transducers registered normal and torque forces produced by the participants' unimanual actions on each of the trays. The defined workspace for the acting hand during the task was limited to an area ∼40 cm in front and 10 cm below the eyes (for the left hand, from centered to ∼20 cm left of a midpoint between the eyes, and respectively to the right for the right hand). Table height was adjusted to each participant enabling them to stand while performing the task. The workspace areas were defined to maximize comfort for the participant while minimizing occlusion of gaze position tracking. Black fabric covered the tabletop and screens mounted on its back and sides to prevent potential distortion of gaze and hand position tracking by reflective light. For the same reason, the participants wore sleeves of black fabric covering their arms down to the wrist level.

### Pegs and peg holes

Each peg consisted of a smooth metal rod (duralumin, diameter, 6 mm; length, 32 mm; weight, 7.6 g). All pegs had uniform color, shape, and weight. Peg holes (diameter, 6.5 mm; depth, 10 mm) were arranged in two rows on a plastic 3D-printed surface (85 × 75 mm). Each row was separated by 23 mm, and peg holes within a row were separated by 13 mm. The front row was 65 mm from the computer monitor.

### Collection trays

The collection trays, one on the left and one on the right side of the peg holes, were 3D-printed plastic concave discs with a 6 cm diameter. The ridge of the disc was aligned with that of the ledge, and the central part of the disc featured a flat surface with a 2 cm radius, situated 7 mm below the outer edge. We designed the tray in this way so that the participants were required to grasp the peg using a precision grip. Before the start of each series of trials, eight pegs were placed flat in the collection tray such that no peg was on top of another peg.

### Gaze position tracking

A gaze tracker (The EyeTribe tracker; sampling rate, 30 Hz) was mounted centered below the workspace facing the eyes of the participant (Fig. 1*B*). For each participant, before the start of the data registration for each round, the gaze tracker was calibrated using the device's standard nine-point calibration procedure. For data analysis, we used the average coordinate registered for the two eyes.

### Hand position tracking

The device for tracking hand motion (Leap Motion Controller; sampling rate, 120 Hz) was mounted centered 40 cm above the workspace (Fig. 1*B*). The setup was optimized to ensure that the entire workspace for peg placement and retrieve movements remained well within the calibrated tracking volume. Before the start of data registration, the device's ability to identify the hand, thumb, and index and middle finger coordinates were confirmed by the experimenter. Participants removed all jewelry and watches prior to the experiment to facilitate tracking. The hand tracking data were aligned with the eye tracking data to a common two-dimensional reference frame in the plane of the computer screen using a custom-made script. This established a unified coordinate system for the sampled hand and gaze coordinates in the plane of the computer screen, positioned 3.5–6.5 cm beyond the first and second row of peg holes from the perspective of the subjects' eyes.

### Force transducers

Force transducers (load cell weight sensors, TZT HX711 Module Electronic Scale Aluminum Alloy Weighting Pressure Sensor, 1 kg, 1,600 Hz) registered normal and torque forces applied to the pegboard tray and two collection trays. Continuous sampling from all devices enabled the registration of task-related force variations to eye and hand positions throughout the task (Fig. 1*C*).

### Additional sensors

Light sensors (sampling rate, 1,600 Hz) were mounted in concave 3D-printed plastic handrests (half spheres; radius, 3 cm). Participants placed their left and right hands in separate handrests at the start of each series. The light sensors were used to confirm the movement onset and offset times for the active hand on each series. The light sensors also served to confirm the stationary status of the passive hand throughout the series.

### Procedure

Each experiment (Fig. 1*D*) comprised multiple blocks defined according to the state of the hand receiving the injections (Experiment 1, nondominant hand; Experiment 2, dominant hand). Experiment 1 comprised two blocks: baseline (no intervention) and anesthesia. Experiment 2 comprised three blocks: baseline, sham injection, and anesthesia. Injections (see below) were performed immediately following Block 1 in Experiment 1 and Blocks 1 and 2 in Experiment 2. Basic sensory and motor capabilities (see below) were evaluated prior to each block and following the final block.

Each block consisted of testing of the dominant and nondominant hands. Each hand was tested in four rounds consecutively. Participants were offered a short break (1–3 min) between rounds. Gaze and hand position tracking calibration was performed prior to the start of each round. Data were continuously recorded over each round.

Each round comprised five series. At the start of each series, the hands were placed on top of handrests equipped with light sensors. Series were divided into placement and retrieval phases. An acoustic signal indicated when participants should begin each phase. During the placement phase, participants moved the pegs, one at a time, from the tray to the peg holes. The collection and delivery of a single peg defined a placement trial. After placing all eight pegs, participants returned the active hand to the handrest. After a 4 s pause, an acoustic signal prompted participants to begin the retrieval phase. During the retrieval phase, participants moved the pegs, one at a time, from the peg holes to the collection tray. The collection and return of a single peg defined a retrieval trial. After returning all the pegs to the collection tray, participants placed the active hand back on the handrest to signal the end of the series.

Participants were instructed to use a precision grip to grasp and place the pegs. No instructions were given regarding how to collect the pegs (other than one at a time), regarding the order of the pegs, or regarding gaze direction (i.e., the participants were free to find their own strategy). If a peg was dropped, participants were instructed to continue by picking up a new peg and leaving the dropped peg for last.

In Experiment 1, each participant performed 1,280 total trials. In Experiment 2, each participant performed 1,920 total trials.

### Digital anesthesia and saline control injections

In both experiments, digital anesthesia was performed by a hand surgeon (author P.F.N.) experienced in regional anesthesia in the hand and arm. We regionally anesthetized the three digits of the hand involved in dexterous manipulation: the thumb, the index finger, and the middle finger. To induce an immediate and persistent digital anesthesia effect, we used a 1:1 mix of the fast-onset local anesthetic lidocaine (1%) and the long duration local anesthetic levobupivacaine (0.5%). A 1.5 ml volume of this local anesthetic mix was injected in each of the addressed fingers with a volar approach through a 27 gauge needle, with the participants in supine posture. For the six participants tested in Experiment 2, isotonic saline injections (1.5 ml volume of 0.9% NaCl isotonic saline) were performed to their dominant hand in the manner described for the digital anesthesia immediately following the completion of the baseline testing blocks.

### Evaluating basic motor and sensory capabilities

Motor and sensory functions of the fingers on the manipulated hand were evaluated at the start of the test sessions (prior to the baseline blocks), prior to the testing blocks that followed the sham and anesthesia injections, and at the end of test sessions (after the anesthesia blocks). Accordingly, the fingers on the nondominant hand were evaluated three times in Experiment 1, and the fingers on the dominant hand were evaluated four times in Experiment 2. These qualitative evaluations were intended as gross characterizations of participants' sensory and motor functions before and after the interventions. We found that participants' range of motion (ROM) for the thumb, index finger, and middle finger were unchanged by the sham and anesthesia injections compared with baseline ROM. In contrast, anesthesia resulted in an extreme loss of sensation for sharp touch in all participants as characterized the static two-point discrimination (2PD) test (measured in the radial and ulnar half of volar fingertip for thumb, index finger, and middle finger). Specifically, all participants had typical 2PD thresholds ranging from 3 to 4 mm prior to the baseline blocks (in Experiments 1 and 2) and after sham injection (in Experiment 2). After anesthesia, we were unable to measure 2PD thresholds which exceeded the maximum testing range of our measurement device (15 mm) in all participants. Thresholds continued to exceed 15 mm when assessed at the end of the anesthesia blocks. There were no adverse effects, and all participants were able to complete the task after digital anesthesia.

### Data curation

From the continuously recorded data, we segmented the placement and retrieval phases into discrete collection, transport, and delivery actions objectively using the normal force signals measured at the pegboard and collection trays. Collection action onset was defined as a change point as detected by the Primed Exact Linear Time (PELT) method (Killick et al., 2012) in the normal force (measured at the collection tray or pegboard), and collection action offset was defined by a change point with a return of normal force to baseline levels. Delivery action onset was defined as a change point in the normal force (measured at the pegboard or collection trays) detected by the PELT method, and delivery action offset was defined by a change point with a return of normal force to baseline levels. Change points were then manually inspected and corrected as needed. Transport actions were defined by each collection offset time and the subsequent delivery onset time. Because the weight of the delivered pegs introduced a consistent and accumulating offset to the force transducer measurements over the series, we computed the offset pattern and detrended this pattern from the entire series such that the forces associated with the start of trial were re-centered to 0. The baseline was defined as the average of the onset and offset points after detrending. In Experiment 1, our system failed to record torque force data on a subset of trials. These failures were only identified after data collection, and these trials were excluded from the torque force analysis.

### Data analysis

Analyses were performed using MATLAB (R2023a) and RStudio (R version 2024.12.0 + 467) on a MacBook Pro running macOS Sonoma 14.6.1. We first characterized the impact of digital anesthesia on the performance speed and forces produced during peg collection, transport, and delivery in order to relate our work to prior studies (Gentilucci et al., 1997; Jenmalm et al., 2000; Augurelle et al., 2003). Our analyses distinguished between trials involving peg collection from the large collection trays with delivery to the small peg holes (precise delivery) and trials with collection from peg holes with delivery to the large collection trays (coarse delivery). This distinction allowed us to test the hypothesis that gaze–hand position relationships depend on the action being performed. To determine the impact of digital anesthesia, we quantified action durations (difference between onset and offset times), peak normal force, and total torque force. We also quantified peg transport durations (time elapsed from collection offset to delivery onset) and hand movement durations without pegs (time elapsed from delivery offset to collection onset). We report times rounded to the nearest hundredth of a millisecond and force to the nearest thousandth of a Newton or Newton meter for normal and torque force, respectively. We defined the forces from the acting hand in downward direction against the tray/peg holes as positive normal forces and computed absolute values to quantify normal force magnitude. To quantify torque force, we similarly computed the absolute value to estimate total force irrespective of direction along the recorded axis.

To determine how digital anesthesia modulated gaze–hand position relationships, we quantified the spatial separation between gaze position and index finger position during peg collection, transport, delivery actions, and during peg-free hand movement. Given the configuration of the pegboard and collection trays, most of the positional variance during task performance was confined to the horizontal axis, so we computed the distance between the gaze position and index finger position (gaze-index separation index, GISI) in the *x*-axis. In separate analyses, we confirmed that positional variance in the *y*-axis was limited and variance in distance metrics computed in two dimensions was dominated by the variance in the horizontal dimension. For collection and delivery actions, GISI was calculated during the time of peak normal force and as a function of normalized progress through the actions defined as the percentage of the full action duration (action progress, AP). To normalize the percentage progress through actions, we split the progress into quartiles using a snapshot of the point coinciding with the quartile based on the range between action onset (0% progress) and offset (100% progress) as demarcated by force plate readings. GISI was similarly calculated as a function of normalized progress over the peg transport and peg-free hand movement actions. Separate analyses were performed for actions extracted from the placement and retrieval trials. To quantify the changes in GISI within a hand, we calculate the difference of GISI between baseline and treatment (ΔGISI). To evaluate how ΔGISI differed between the dominant and nondominant hand, we computed the difference of the ΔGISI metric between hands (ΔΔGISI). This analysis approach enables us to quantify the effect of our interventions while accounting for potential learning or fatigue effects related to the passage of time.

We statistically assessed how force, duration, and GISI were modulated according to hand and condition block (baseline, sham, anesthesia blocks) using linear mixed-effect models from the *lme4* package (Bates et al., 2015). To test modulation of force, duration, and GISI at peak force, we included block (Experiment 1, baseline vs postanesthesia; Experiment 2, baseline vs sham vs anesthesia) and hand (dominant vs nondominant) as fixed effects. To account for subject-specific variance and learning effects, we included series nested within subject as a random effect on the slope. Thus, the model for any dependent variable (DV) took the following form:

DV=Block*Hand+(1|Subject/Round).

We similarly tested modulation of GISI as a function of normalized progress by including percentage progress through the action as an additional fixed effect. The model took the following base form:

GISI=Block*Hand*Progress+(1+Block*Hand*Progress|Subject)+(0+Round|Subject).

We used the *car* package (Fox et al., 2001) to estimate the statistical significance for all fixed effects (Type 2 Wald *χ*^2^ tests). Post hoc tests were calculated using the *emmeans* package (Searle et al., 1980; Lenth, 2017), which applies multiple-comparison correction (Tukey's test). Because of the large number of trials across participants, we followed the package recommendation to use the asymptotic method for degrees of freedom for the post hoc tests.

To statistically assess for an effect of learning, we again modeled the hand and block as fixed effects along with the series within a block (Series_Block_). Series_Block_ accounted for previous experience within a block, thus examining learning through a given condition. We applied this model to force duration and GISI at peak force as DVs to determine the slope as a measure of learning:

$$\mathrm{DV}=\mathrm{Block}\;*\;\mathrm{Hand}\;*\;\mathrm{Serie}s_{\mathrm{Block}}+(1|\mathrm{Subject}/\mathrm{Round}).$$

The trend (slope) of the DV was calculated with the emtrends function from the emmeans package to evaluate how the response changes with respect to Series_Block_.

For a complementary analysis of the spatiotemporal relationship between gaze and hand positions, we computed the cross-correlation between gaze and hand position using an entire series of eight pegs within a specific task phase after downsampling the hand data to 30 Hz to match the sample rate of the gaze data. In particular, we were interested in computing the lag (temporal offset) between the gaze and hand position time series. Temporal offset was modeled with the following equation:

Offset=Block*Hand*Phase+(1+Block*Hand*Phase|Subject).

From all models, we simplified the fixed effects, random effects, or both to fit the most comprehensive model that still converged. The final form of the model for each analysis is described in the code provided as Extended Data.

### Code accessibility

The code/software described in the paper is freely available online at https://github.com/YauLab/NumbPeg (Extended Data 1).

10.1523/ENEURO.0487-23.2025.d1 Extended Data The file Code_and_Data.zip includes all code and data used to perform the analyses presented in this manuscript. The .R scripts contain the packages used and the filenames corresponding to the data and the structure of each model. Download Extended Data, ZIP file.

## Results

### Digital anesthesia alters action durations and force production

We briefly summarize the effects of digital anesthesia on performance during a dexterous motor task (for summarized description of task, see Fig. 1) before presenting the results related to the gaze–hand position relationship, the primary focus of our study. The task was split into two phases: placement or retrieval of pegs. Within each phase, we defined four actions using the force signals measured at the collection trays and pegboard: peg-free hand movement, peg collection, peg transport, and peg delivery. This delineation allowed us to analyze the actions of the tasks in terms of the precision required: coarse collection and delivery involving the collection trays or precise collection and delivery involving the pegboard.

In Experiments 1 and 2, we found that digital anesthesia systematically and reliably altered performance on the dexterous motor task (Extended Data). While participants successfully completed all actions in the placement phase (Extended Data Figs. 2-1, 2-2, 4-1, 4-2) and retrieval phase (Extended Data Figs. 3-1, 3-2, 5-1, 5-2), digital anesthesia resulted in significant changes in the time required to complete the actions and the forces produced by the actions. These results reveal that the acute loss of somatosensory feedback slows motor performance and the capacity to regulate force, consistent with previous studies in deafferented patients (Cuadra et al., 2019). Importantly, saline injections did not induce the same behavioral changes (Extended Data Figs. 4-1, 4-2, 5-1, 5-2), indicating that the altered performance was specifically attributable to the loss of tactile sensation.

### Experiment 1: anesthesia modulates eye–hand coordination over phases

We first sought to determine how the loss of sensory feedback on the hand-modulated eye–hand coordination at the level of series which comprised placement and retrieval phases. Given the cyclical nature of the actions within a phase (sequential engagement of eight pegs), we reasoned that the temporal offset computed through cross-correlation analysis would provide a metric capturing the relationship between the gaze and hand positions within a phase. We compared the temporal offset metrics computed from each phase completed before (B1) and after digital anesthesia (B2) to determine the general modulatory effects associated with the loss of sensation. Without anesthesia, gaze position routinely preceded that of the hand in the placement phase (158.7  31.8 ms), consistent with the guidance role of vision. With anesthesia, the offset between the gaze and hand was substantially smaller (73.3 ± 31.8 ms). A model accounted for these patterns with a significant block * hand interaction ( $X_{1}^{2}$ = 8.0; *p* = 0.005). Post hoc tests revealed that the change in offset for the treated hand differed significantly from the change in offset for the untreated hand (ΔΔoffset = 58 ± 21.2 ms; *t*_(788)_ = 2.7; *p* = 0.006). Notably, anesthesia did not similarly modulate the gaze–hand relationship in the retrieval phase (ΔΔoffset = 26.7 ± 21.2 ms; *t*_(788)_ = 1.3, *p* = 0.2). The absence of an effect in the retrieval phase may be related to the different gaze demands compared with the placement phase. To address this possibility, we next characterized gaze–hand relationships during each action.

### Experiment 1: eye–hand coordination in the placement phase is altered by digital anesthesia

Having demonstrated that digital anesthesia modulated performance times and forces generated in the pegboard task, we focused on how somatosensory feedback loss impacted the spatiotemporal relationship between participants' gaze and hand positions during the task. When grasping and transporting objects with intact senses, vision serves primarily to guide planning of subsequent hand movements (Johansson et al., 2001), so gaze position typically leads hand position in space. In this mode, somatosensory signals provide feedback regarding the physical interactions between the hand and grasped object (Johansson and Flanagan, 2009). In the context of our pegboard task, somatosensory feedback would signal the forces applied during peg collection, successful peg grasping and delivery, and stable peg handling during transport. With somatosensory feedback loss, we hypothesized that vision would be recruited to provide substitute feedback about the interactions between the hand and grasped object. Accordingly, we predicted that gaze position would be more closely tied to hand position after digital anesthesia.

**Figure 2.**
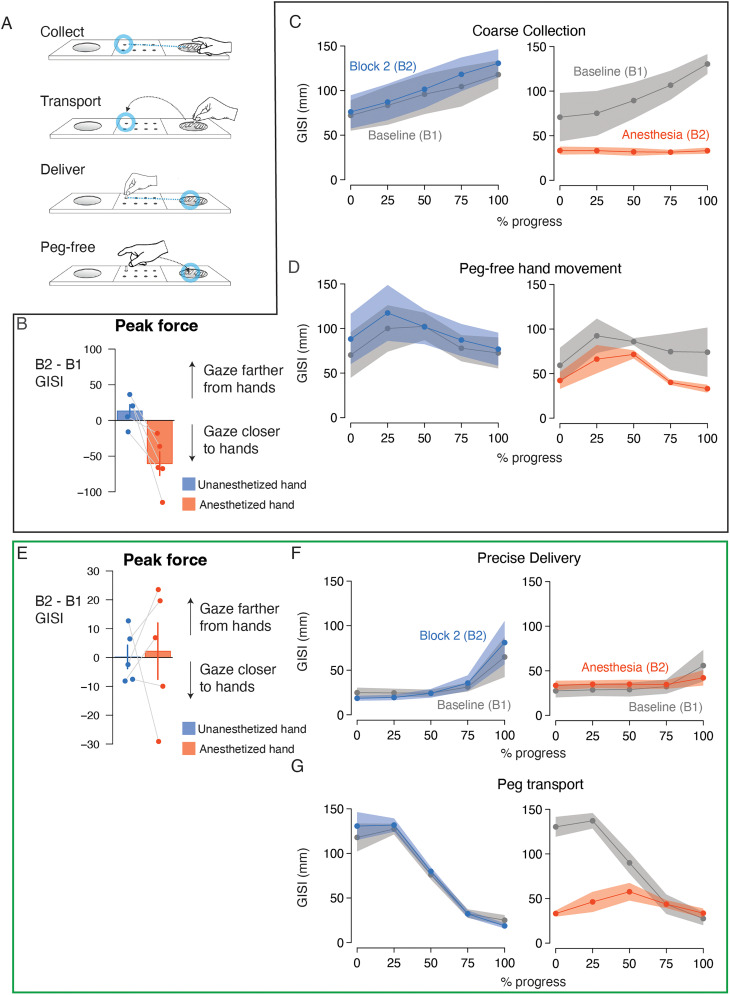
Experiment 1: gaze more closely tracked the hands in the placement phase following anesthesia treatment. ***A***, Schematic of the task during the placement phase in Experiment 1 divided into the constitutive actions. ***B***, Bar plots showing the mean change in GISI at peak force during coarse collection between Block 1 (preanesthesia) and Block 2 (postanesthesia) with the dominant (blue) and nondominant (red) hands. The error bar indicates SEM. Connected markers indicate data from individual participants. ***C***, Lines and markers indicate mean GISI (average of participant-level means) calculated as a function of progress (%) through the coarse collection action with the dominant hand (left plot) and nondominant hand (right plot). The shaded region indicate SEM. Gray data indicate GISI in the baseline blocks. Blue data indicate GISI for the dominant hand (control) in Block 2. Red data indicate GISI for the nondominant (anesthetized) hand in Block 2. ***D***, Lines and markers indicate mean GISI calculated as a function of progress (%) through the peg-free hand movement action. Conventions as in ***C***. ***E***, Bar plots showing the mean change in GISI at peak force during precise delivery. Conventions as in ***B***. ***F***, Lines and markers indicate mean GISI calculated as a function of progress (%) through the precise delivery action. Conventions as in ***C***. ***G***, Lines and markers indicate mean GISI calculated as a function of progress (%) through the peg transport action. Conventions as in ***C***. Force data for the placement phase of Experiment 1 is shown in Extended Data Figure 2-1. Summary statistics are provided in Extended Data Figure 2-2. Statistics of potential learning effects are provided in Extended Data Figure 2-3.

Having split the task into placement and retrieval phases, we further subdivided the actions within each phase to collection and delivery of the peg (Fig. 2*A*) which involved physical interactions with the tray and pegboard. The phases also comprised movement actions—peg-free hand movement and peg transport—directly preceding the peg collection and delivery actions, respectively. We first examined GISI (see Materials and Methods) at the time of peak normal force production during peg collection, reasoning that participants were maximally engaged with the pegs at this time. A model revealed a significant block * hand interaction ( $X_{1}^{2}$ = 187.7; *p* < 10^−16^). Post hoc tests (Fig. 2*B*) revealed a significant decrease in GISI (ΔGISI) after digital anesthesia within the treated hand (ΔGISI = −57.6 ± 3.7 mm; *z* = −15.7; *p* < 0.0001). A comparison between ΔGISI for the treated and untreated hands also revealed significant differences (ΔΔGISI = −70.6 ± 5.2 mm; *z* = −13.7; *p* < 0.0001). Given that the time of peak normal force varied across trials throughout coarse collection, we additionally modeled GISI as a function of progress through coarse collection (Fig. 2*C*). GISI increased monotonically through the action for the untreated hand in B1 and B2. GISI similarly increased monotonically through the action for the treated hand in B1 but remained constant following anesthesia treatment in B2. The model captured these patterns as a significant block * hand * progress interaction ( $X_{4}^{2}$ = 178.2; *p* < 10^−16^). Post hoc tests captured the increasing across-hand differences in ΔGISI through the action (onset, ΔΔGISI = −39.6 ± 19.0 mm; *z* = −2.1; *p* = 0.04; AP_25_, ΔΔGISI = −44.4 ± 19.0 mm; *z* = −2.3; *p* = 0.02; AP_50_, ΔΔGISI = −62.9 ± 19.0 mm; *z* = −3.3; *p* = 0.001; AP_75_, ΔΔGISI = −90.0 ± 19.0 mm; *z* = −4.7; *p* < 0.0001; offset, ΔΔGISI = −112.1 ± 19.0 mm; *z* = −5.9; *p* < 0.0001). These data suggest that participants were typically less reliant on gaze to accomplish coarse collection and quicker to look ahead to the next location in control conditions. In contrast, participants looked closer at their hands and maintained this gaze–hand relationship during coarse collection following the loss of somatosensory feedback.

10.1523/ENEURO.0487-23.2025.f2-1 Figure 2-1 Force results for Experiment 1 during the placement phase. Bar graphs of force data during the placement phase, i.e., peg pick-up from tray (coarse collection) and delivery to peg-holes (precise delivery) of experiment 1. (A-C) From coarse collection, bar graphs across all participants and conditions of duration of force application in the collection tray, peak normal force produced in the collection tray, and total torque force in the collection tray. (D-F) From precise delivery, bar graphs across all participants and conditions of duration of force application in the peg-hole, peak normal force produced in the peg-hole, and total torque force in the peg-hole. (G-H) Bar graphs depicting, for all trials sorted by condition, time elapsed between peg transport (completion of peg collection and initiating peg delivery for all trials sorted by condition), and peg-free hand movement (completion of peg delivery and initiating collection of the next peg). Bar graphs represent the mean with individual data points representing individual subjects. Error bar represents s.e.m. Download Figure 2-1, TIF file.

10.1523/ENEURO.0487-23.2025.f2-2 Figure 2-2 Statistical summary of the GLM of the force data for Experiment 1 during the placement phase. (A-C) Statistical results table for force data related to coarse collection during the placement phase. Refer to (A-C) in Extended Data Figure 2-1 for visualized data. (D-F) Statistical results table for force data related to precise delivery during the placement phase. Refer to (D-F) in Extended Data Figure 2-1 for visualized data. (G-H) Statistical results table for transport and peg-free hand movement durations during the placement phase. Refer to (G-H) in Extended Data Figure 2-1 for visualized data. Download Figure 2-2, TIF file.

**Figure 3.**
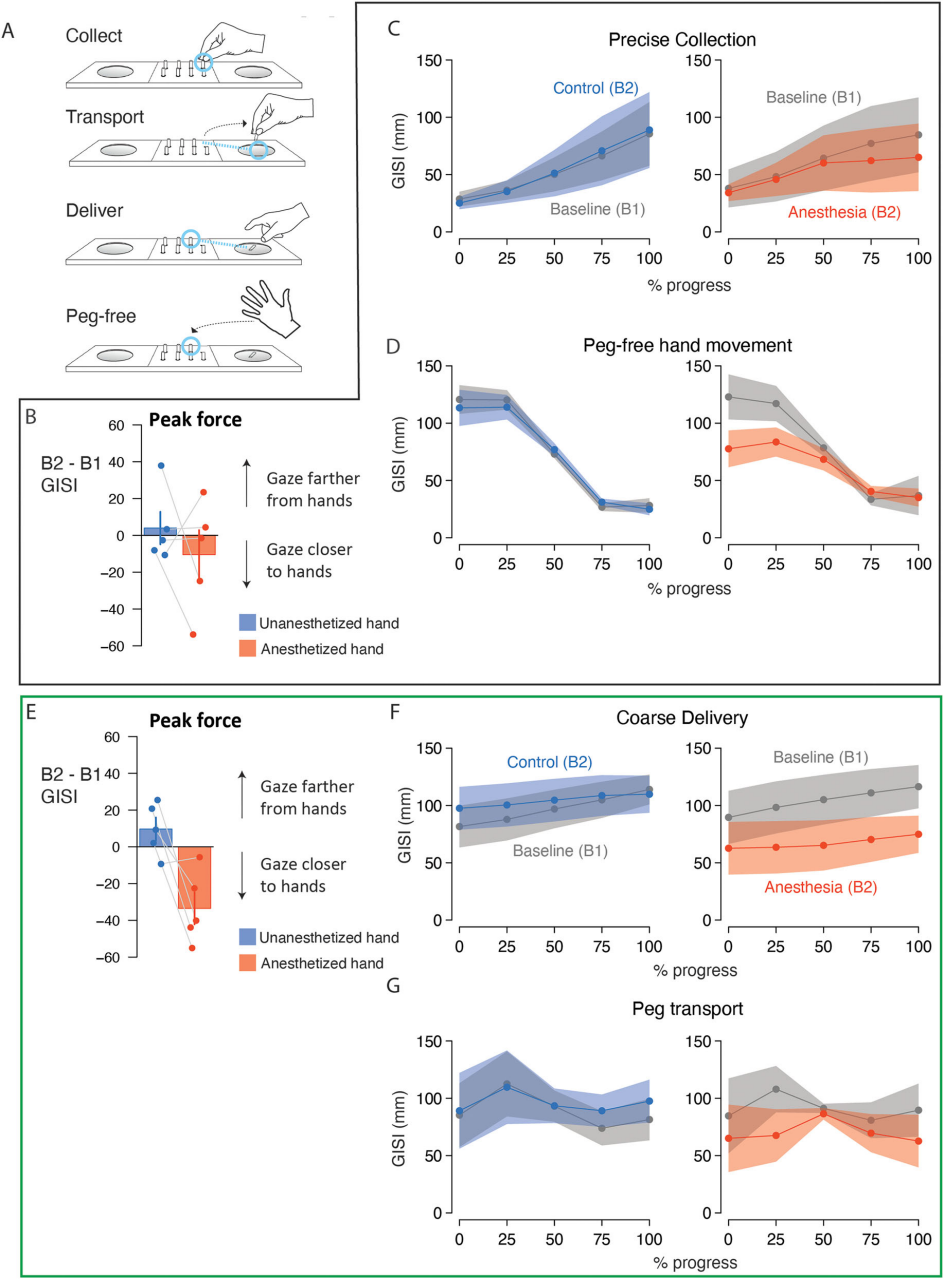
Experiment 1: gaze more closely tracked the hands in the retrieval phase after anesthesia treatment. ***A***, Schematic of the task during the retrieval phase in Experiment 1 divided into the constitutive actions. ***B***, Bar plots showing the mean change in GISI at peak force during precise collection between Block 1 (preanesthesia) and Block 2 (postanesthesia) with the dominant (blue) and nondominant (red) hands. The error bar indicate SEM. Connected markers indicate data from individual participants. ***C***, Lines and markers indicate mean GISI (average of participant-level means) calculated as a function of progress (%) through the precise collection action with the dominant hand (left plot) and nondominant hand (right plot). The shaded region indicates SEM. Gray data indicate GISI in the baseline blocks. Blue data indicate GISI for the dominant hand (control) in Block 2. Red data indicate GISI for the nondominant (anesthetized) hand in Block 2. ***D***, Lines and markers indicate mean GISI calculated as a function of progress (%) through the peg transport action. Conventions as in ***C***. ***E***, Bar plots showing the mean change in GISI at peak force during coarse delivery. Conventions as in ***B***. ***F***, Lines and markers indicate mean GISI calculated as a function of progress (%) through the coarse delivery action. Conventions as in ***C***. ***G***, Lines and markers indicate mean GISI calculated as a function of progress (%) through the peg transport action. Conventions as in ***C***. Force data for the retrieval phase of Experiment 1 are shown in Extended Data Figure 3-1. Summary statistics are provided in Extended Data Figure 3-2. Statistics of potential learning effects are provided in Extended Data Figure 3-3.

10.1523/ENEURO.0487-23.2025.f2-3 Figure 2-3 Evaluation of potential learning effects on force duration and GISI at peak force during the placement phase in Experiment 1. Traces indicate average force duration and GISI at peak force as a function of series with the anesthetized (red) and unanesthetized (blue) hands during each action. Changes over series were evaluated separately for Block 1 (baseline) and Block 2 (anesthesia). Tables indicate significant and non-significant slopes from linear model fits. For this analysis, we predicted that learning effects – if present – would reflect increased efficiency in task performance, such that force duration would decrease across series and GISI would increase. Significant linear trends were variable, tended to be small, and were inconsistent with learning. Download Figure 2-3, TIF file.

Given the adaptations of eye–hand coordination when interacting with a peg, we next asked whether these adjustments also occur in actions that did not involve peg manipulation. We examined gaze–hand position relationships during all peg-free hand movement actions leading up to coarse collection (Fig. 2*D*; i.e., hand movements after peg delivery and prior to peg collection). Given that nothing was held in the hand during this action, we did not predict differences in gaze–hand relationship related to anesthesia for these events. Contrary to our prediction, a model revealed a significant block * hand * progress interaction ( $X_{4}^{2}$ = 27.1; *p* = 10^−5^; Fig. 2*D*). Post hoc tests revealed significant differences in ΔGISI for the treated and untreated hands throughout the movement action (onset, ΔΔGISI = −36.8 ± 5.1 mm; *z* = −7.2; *p* < 0.001; AP_25_, ΔΔGISI = −45.8 ± 5.1 mm; *z* = −9.0; *p* < 0.0001; AP_50_, ΔΔGISI = −11.4 ± 5.1 mm; *z* = −2.2; *p* = 0.02; AP_75_, ΔΔGISI = −38.8 ± 5.1 mm; *z* = −7.6; *p* < 0.0001; offset, ΔΔGISI = −38.6 ± 5.1 mm; *z* = −7.6; *p* < 0.0001). These results indicated that the reduced separation in gaze–hand position during the anesthesia condition was maintained throughout the hand movement action. The fact that gaze was more closely anchored to the hand during peg-free hand movements indicates that the compensatory changes in eye–hand coordination are not specific to hand actions involving object manipulation.

We then examined gaze–hand position relationships during precise delivery when participants were tasked with placing the peg into a small hole on the pegboard. We reasoned that this action required more precision and a higher degree of eye–hand coordination compared with the other actions even with intact somatosensation. Accordingly, we predicted that gaze–hand relationships would be relatively unchanged comparing B2 and B1 for both hands. Consistent with our prediction, the block * hand interaction failed to reach significance ( $X_{1}^{2}$ = 0.29; *p* = 0.59) for GISI differences at the time of peak normal force production (Fig. 2*E*). Visual inspection of GISI dynamics over the delivery action (Fig. 2*F*) indicated subtle differences between the treated and untreated hands: GISI increased at the end of the delivery action with both hands prior to treatment and with the untreated hand in B2, while GISI remained low with the anesthetized hand. These patterns were captured by a significant block * hand * progress interaction ( $X_{4}^{2}$ = 113.0; *p* < 10^−16^), driven by ΔGISI difference between treated and untreated hands only during the end of the delivery action (ΔΔGISI = −29.9 ± 9.2 mm; *z* = −3.3; *p* = 0.001). These results revealed that performance of precise delivery under all conditions required gaze recruitment; however, gaze position was released more quickly following peg delivery in conditions with reliable somatosensation compared with the anesthesia condition. The loss of somatosensory feedback appeared to increase participants' reliance on vision to complete precise peg delivery.

Lastly, we examined gaze–hand position relationships during peg transport leading to precise delivery (Fig. 2*G*). At the start of this transport action, participants look toward the pegboard while they maintain their hold on the peg with intact somatosensation resulting in a relatively large GISI. In the anesthesia condition, participants fixed their gaze on their hand, resulting in a small GISI that remained relatively unchanged throughout the action. A model accounted for these patterns with a significant block * hand * progress interaction ( $X_{4}^{2}$ = 1,133.6; *p* < 10^−16^). Post hoc tests revealed significant hand differences at the onset of the transport action (ΔΔGISI = −109.7 ± 9.8 mm; *z* = −11.2; *p* < 0.0001) and the first half of the transport action (AP_25_, ΔΔGISI = −94.8 ± 9.8 mm; *z* = −9.7; *p* < 0.0001; AP_50_, ΔΔGISI = −35.5 ± 9.8 mm; *z* = −3.6; *p* = 0.0003). Together, these results support our hypothesis that vision, which typically precedes and guides hand movements, was recruited to monitor the status of the grasped peg during transport following somatosensory feedback loss.

In sum, these analyses revealed the effect of digital anesthesia on eye–hand coordination during collection, transport, and delivery actions during the placement phase: Gaze tracked more closely with the hand following the loss of somatosensory feedback. GISI changes related to digital anesthesia were obvious during coarse collection and peg transport events, implying that vision was recruited to support feedback mechanisms typically governed by touch. GISI modulation during precise delivery may be more subtle because this action typically required more visual support. Notably, the closer gaze–hand relationship during peg-free hand movements suggests a general compensatory mechanism in which vision becomes spatially yoked to the hand even in the absence of object manipulation.

### Experiment 1: eye–hand coordination in the retrieval phase is altered by digital anesthesia

To address whether the changes in gaze–hand coordination were context dependent, we also analyzed gaze–hand relationships during the retrieval phase of the task, where peg collection required more precision and peg delivery was coarser than in the placement phase (Fig. 3*A*). Analysis of the peg-free hand movement and transport actions additionally enabled us to evaluate whether gaze–hand compensatory changes extended to movement actions and required peg manipulation.

10.1523/ENEURO.0487-23.2025.f3-1 Figure 3-1 Force results for Experiment 1 during the retrieval phase. Bar graphs of force data during the retrieval phase, i.e., peg pick-up from tray (coarse collection) and delivery to peg-holes (precise delivery) of experiment 1. (A-C) From coarse collection, bar graphs across all participants and conditions of duration of force application in the collection tray, peak normal force produced in the collection tray, and total torque force in the collection tray. (D-F) From precise delivery, bar graphs across all participants and conditions of duration of force application in the peg-hole, peak normal force produced in the peg-hole, and total torque force in the peg-hole. (G-H) Bar graphs depicting, for all trials sorted by condition, time elapsed between peg transport (completion of peg collection and initiating peg delivery for all trials sorted by condition), and peg-free hand movement (completion of peg delivery and initiating collection of the next peg). Bar graphs represent the mean with individual data points representing individual subjects. Error bar represents s.e.m. Download Figure 3-1, TIF file.

10.1523/ENEURO.0487-23.2025.f3-2 Figure 3-2 Statistical summary of the GLM of the force data for Experiment 1 during the retrieval phase. (A-C) Statistical results table for force data related to precise collection during the retrieval phase. Refer to (A-C) in Extended Data Figure 3-1 for visualized data. (D-F) Statistical results table for force data related to coarse delivery during the retrieval phase. Refer to (D-F) in Extended Data Figure 3-1 for visualized data. (G-H) Statistical results table for transport and peg-free hand movement durations during the retrieval phase. Refer to (G-H) in Extended Data Figure 3-1 for visualized data. Download Figure 3-2, TIF file.

10.1523/ENEURO.0487-23.2025.f3-3 Figure 3-3 Evaluation of potential learning effects on force duration and GISI at peak force during the retrieval phase in Experiment 1. Traces indicate average force duration and GISI at peak force as a function of series with the anesthetized (red) and unanesthetized (blue) hands during each action. Changes over series were evaluated separately for Block 1 (baseline) and Block 2 (anesthesia). Tables indicate significant and non-significant slopes from linear model fits. Download Figure 3-3, TIF file.

To quantify eye–hand coordination during precise collection, we first evaluated gaze–hand relationships when participants produced the maximum force on the pegboard (Fig. 3*B*). A model captured a modest but significant reduction in ΔGISI during peak force application in the treated hand compared with the untreated hand with a block * hand interaction ( $X_{1}^{2}$ = 10.1; *p* = 0.002; ΔΔGISI = −14.4 ± 4.5 mm; *z* = −3.2; *p* = 0.002). Across the precise collection action (Fig. 3*C*), GISI for both hands during B1 and B2 initially exhibited similar increases; however, the separation between the gaze and anesthetized hand leveled off during the second half of the collection action, while the separation continued to grow in the other conditions. The model accounted for this pattern with a significant block * hand * progress interaction ( $X_{4}^{2}$ = 24.5; *p* = 10^−5^). Although ΔΔGISI did not differ significantly in any specific action quartile, ΔGISI for the treated hand tended to be larger than that for the untreated hand at the end of the precise collection (ΔΔGISI = −23.0 ± 14.1 mm). A potential explanation for the more subtle anesthesia effects on precise collection may be that this action required more gaze involvement even with intact somatosensation. Indeed, GISI values during precise collection (56.2 ± 4.4 mm) were significantly lower (*t*_(30.9)_ = 2.6; *p* = 0.01) compared with GISI values during coarse collection (78.8 ± 7.4 mm; Fig. 2*C*).

The peg-free hand movement actions in retrieval phases were generally characterized by an initial large separation in gaze–hand position that decreased over the movement action (Fig. 3*D*). This pattern is consistent with the notion that precise collection is a visually guided process in which participants initially direct their gaze to the pegboard to localize the target peg before they subsequently moved their hand to retrieve the peg. Although visual inspection of the data revealed that gaze and hand positions converge at the pegboard in all conditions, a smaller separation between participants' gaze and their anesthetized hands during the early portion of the movement action was also apparent. These patterns were captured by a significant block * hand * progress interaction ( $X_{4}^{2}$ = 103.1; *p* < 10^−16^). Post hoc tests revealed significant differences between the treated and untreated hand during the initial movement intervals (onset, ΔΔGISI = −37.6 ± 8.2 mm; *z* = −4.6; *p* < 0.0001; AP_25_, ΔΔGISI = −27.27 ± 8.2 mm; *z* = −3.3; *p* = 0.0009). These results indicate that participants directed their gaze closer to their hands following the loss of somatosensory feedback during hand movements preceding precise collection, consistent with the results in the peg-free hand movement action during coarse collection. These results were inconsistent with the hypothesis of a compensatory eye–hand coordination process being event-specific and dependent on interactions with objects.

We next compared gaze–hand position relationship during coarse delivery. During peak force production, ΔGISI differed significantly between the treated and untreated hand (block * hand interaction, $X_{1}^{2}$ = 99.5; *p* < 10^−16^; Fig. 3*E*). Over the duration of the coarse delivery action (Fig. 3*F*), GISI tended to be relatively stable with a minor increase in all conditions; however, GISI was notably lower for the treated hand in B2. Accordingly, a model revealed a significant block * hand interaction ( $X_{1}^{2}$ = 506. 9; *p* < 10^−16^), but the block * hand * progress interaction ( $X_{4}^{2}$ = 3. 5, *p* = 0.48) failed to achieve significance. The post hoc test on the block * hand interaction confirmed that ΔGISI for the anesthetized hand was significantly lower compared with the nonanesthetized hand throughout coarse delivery (ΔΔGISI = 43.7 ± 1.9 mm; *z* = 22.5; *p* < 0.0001). These results demonstrate that vision was recruited to compensate for the loss of somatosensory feedback when participants returned the pegs to the large trays.

Lastly, we evaluated anesthesia effects on gaze–hand relationships during peg transport preceding coarse delivery. Given our hypothesis that vision is recruited to provide object manipulation feedback when touch is lost, we predicted that gaze would be more closely tied to the hand during peg transport with digital anesthesia compared with conditions with intact touch. Consistent with this prediction, we observed reduced separation between participants' gaze and the anesthetized hand (Fig. 3*G*). This pattern was captured by a significant block * hand * progress interaction ( $X_{4}^{2}$ = 35.1; *p* = 10^−7^). Post hoc tests revealed significant ΔΔGISI differences early and late in the transport action (onset, ΔΔGISI = −23.5 ± 8.6 mm; *z* = −2.7; *p* = 0.006; AP_25_, ΔΔGISI = −37.9 ± 8.6 mm; *z* = −4.4; *p* < 0.0001; AP_75_, ΔΔGISI = −26.1 ± 8.6 mm; *z* = −3.0; *p* = 0.002; offset, ΔΔGISI = −42.4 ± 8.6 mm; *z* = −4.9; *p* < 0.0001). These collective results indicate a tighter gaze–hand relationship during peg transport following somatosensory feedback loss.

### Experiment 2: anesthesia modulates eye–hand coordination over phases

In Experiment 1, the nondominant hand received digital anesthesia in all participants. Moreover, behavior with the anesthetized hand was compared only against behavior with the untreated, dominant hand. Accordingly, the task performance and gaze behavior changes could be attributable simply to an increased salience of the nondominant hand because it received an injection rather than reflecting the loss of sensation on that hand. To address this potential confound, we performed a second experiment with a new group of participants. In Experiment 2, participants performed the pegboard task with their dominant hand under three conditions: no treatment (B1), injection with saline (B2), and injection with anesthesia (B3). The nondominant hand was tested during B1, B2, and B3 without treatment to account for the passage of time and potential learning effects. This design explicitly (1) accounts for hand dominance (comparing with Experiment 1), (2) provides an internal control (comparing sham vs anesthesia in Experiment 2), and controls for block (comparing Experiment 1 anesthesia condition with Experiment 2 sham condition).

Just as we did for Experiment 1, we first assessed whether anesthesia modulated gaze–hand relationships at the temporal scale of phases. Consistent with Experiment 1 results, smaller offsets were observed for the anesthetized hand compared with the other conditions. A model captured this effect with a significant block * hand interaction ( $X_{2}^{2}$ = 25.4; *p* = 10^−6^). Post hoc tests revealed that the offset change for the treated hand differed significantly from the offset change for the untreated hand (ΔΔoffset = 100.0 ± 21.6 ms; *t* = 4.6; *p* < 0.0001). In contrast, anesthesia did not significantly modulate gaze–hand relationships during the retrieval phase (ΔΔoffset = 31.67 ± 21.6 ms; *t* = 1.5; *p* = 0.31). These collective results are consistent with the offset results in Experiment 1.

### Experiment 2: eye–hand coordination in the placement phase is altered by digital anesthesia

**Figure 4.**
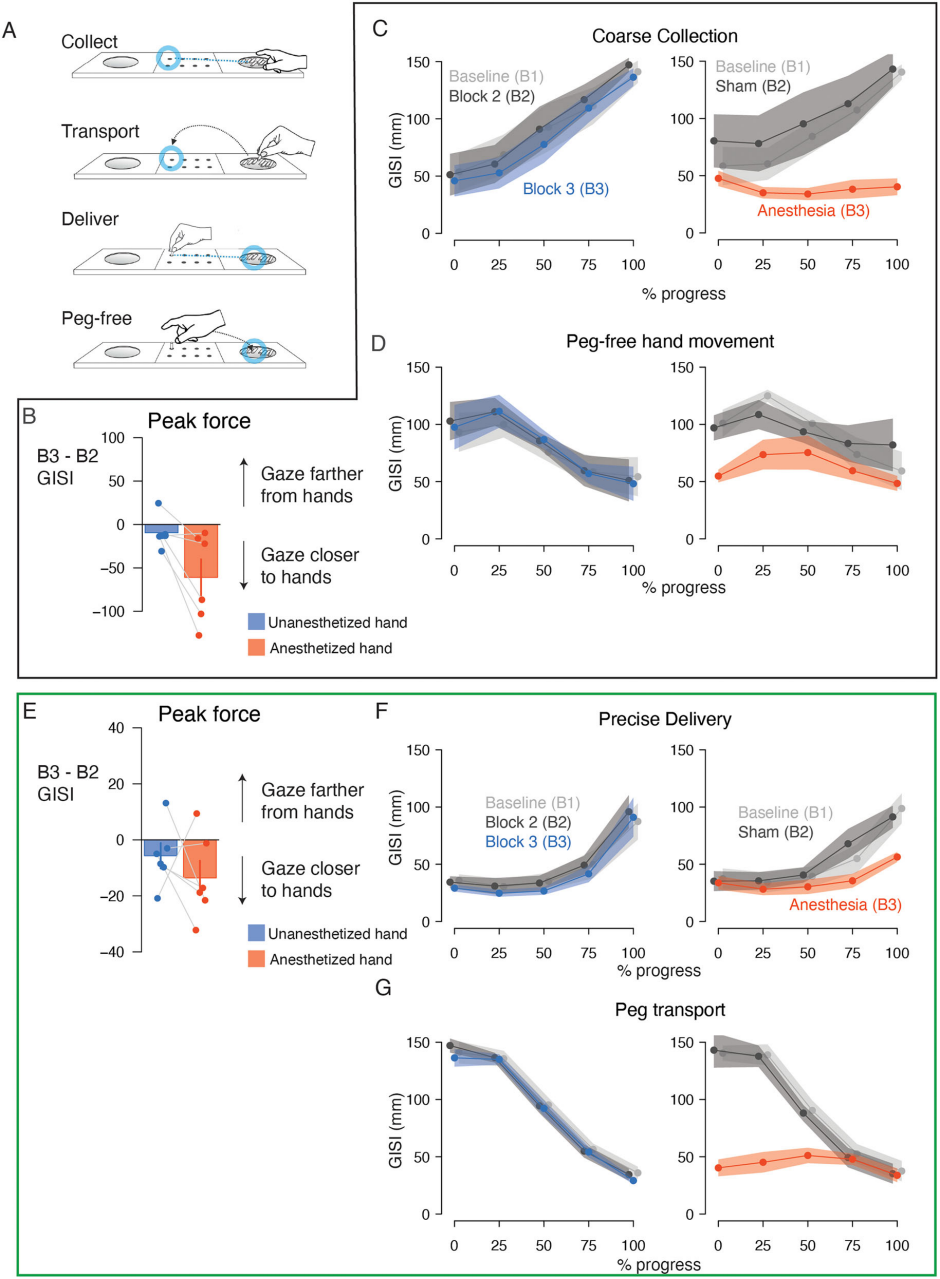
Experiment 2: gaze more closely tracked the hands in the placement phase following anesthesia treatment. ***A***, Schematic of the task during the placement phase in Experiment 2 divided into the constitutive actions. ***B***, Bar plots showing the mean change in GISI at peak force during coarse collection between Block 2 (sham) and Block 3 (postanesthesia) with the nondominant (blue) and dominant (red) hands. The error bar indicates SEM. Connected markers indicate data from individual participants. ***C***, Lines and markers indicate mean GISI (average of participant-level means) calculated as a function of progress (%) through the coarse collection action with the nondominant hand (left plot) and dominant hand (right plot). The shaded region indicates SEM. Light gray data indicate GISI in the baseline blocks. Dark gray data indicate GISI in Block 2 for the nondominant hand and dominant hand (sham). Blue data indicate GISI for the nondominant hand in Block 3. Red data indicate GISI for the dominant (anesthetized) hand in Block 3. ***D***, Lines and markers indicate mean GISI calculated as a function of progress (%) through the peg-free hand movement action. Conventions as in ***C***. ***E***, Bar plots showing the mean change in GISI at peak force during precise delivery. Conventions as in ***B***. ***F***, Lines and markers indicate mean GISI calculated as a function of progress (%) through the precise delivery action. Conventions as in ***C***. ***G***, Lines and markers indicate mean GISI calculated as a function of progress (%) through the peg transport action. Conventions as in ***C***. Force data for the placement phase of Experiment 2 is shown in Extended Data Figure 4-1. Summary statistics are provided in Extended Data Figure 4-2. Statistics of potential learning effects are provided in Extended Data Figure 4-3.

During the coarse collection action (Fig. 4), we found a significant block * hand interaction on the separation of the gaze and hand at the time of peak force production ( $X_{2}^{2}$ = 124.5; *p* < 10^−16^) contrasting the results from testing after sham injection (B2) compared with testing after anesthesia injection (B3). A post hoc test (Fig. 4*B*) revealed that the B3–B2 difference (ΔGISI) with the dominant (treated) hand was significantly larger than the difference with the nondominant hand (ΔΔGISI = 51.4 ± 7.8 mm; *z* = 10.7; *p* < 0.0001). Considering how gaze–hand separation varied over the coarse collection action (Fig. 4*C*), we observed that the separation increased monotonically through the action when testing with intact sensation for both hand testing (i.e., during B1 and B2). Notably, the gaze–hand separation remained relatively constant during the action with digital anesthesia, suggesting that gaze position was relatively fixed on hand position (Fig. 4*C*, right). These patterns were captured by a significant block * hand * progress interaction ( $X_{8}^{2}$ = 433.12; *p* < 10^−16^). Post hoc tests revealed significant gaze–hand separation differences comparing the treated hand during B3 compared with B1 (AP_50_, ΔΔGISI = −28.1 ± 4.5 mm; *z* = −6.2; *p* < 0.0001; AP_75_, ΔΔGISI = −65.7 ± 4.5 mm; *z* = −14.5; *p* < 0.0001; offset, ΔΔGISI = −100.0 ± 4.5 mm; *z* = −22.1; *p* < 0.0001) and B2 (onset, ΔΔGISI = −23.2 ± 4.5 mm; *z* = −5.1; *p* < 0.0001; AP_25_, ΔΔGISI = −28.0 ± 4.5 mm; *z* = −6.2; *p* < 0.0001; AP_50_, ΔΔGISI = −39.1 ± 4.5 mm; *z* = −8.6; *p* < 0.0001; AP_75_, ΔΔGISI = −65.1 ± 4.5 mm; *z* = −14.4; *p* < 0.0001; offset, ΔΔGISI = −100.0 ± 4.5 mm; *z* = −21.0; *p* < 0.0001) for most progress points. Notably, a block * hand interaction ( $X_{2}^{2}$ = 654.6; *p* < 10^−16^) and follow-up post hoc analysis also revealed modest but significant gaze–hand coordination changes after sham injection (B2–B1; ΔΔGISI = 13.6 ± 2.0 mm; *z* = 6.7; *p* < 0.0001) but with increased gaze–hand separation compared with the no injection condition. Accordingly, the dramatic reduction in gaze–hand separation following digital anesthesia occurred despite the effects observed from the sham intervention. These anesthesia results are consistent with the coarse collection results in Experiment 1.

10.1523/ENEURO.0487-23.2025.f4-1 Figure 4-1 Force results for Experiment 2 during the placement phase. Bar graphs of force data during the placement phase, i.e., peg pick-up from tray (coarse collection) and delivery to peg-holes (precise delivery) of experiment 2. (A-C) From coarse collection, bar graphs across all participants and conditions of duration of force application in the collection tray, peak normal force produced in the collection tray, and total torque force in the collection tray. (D-F) From precise delivery, bar graphs across all participants and conditions of duration of force application in the peg-hole, peak normal force produced in the peg-hole, and total torque force in the peg-hole. (G-H) Bar graphs depicting, for all trials sorted by condition, time elapsed between peg transport (completion of peg collection and initiating peg delivery for all trials sorted by condition), and peg-free hand movement (completion of peg delivery and initiating collection of the next peg). Bar graphs represent the mean with individual data points representing individual subjects. Error bar represents s.e.m. Download Figure 4-1, TIF file.

10.1523/ENEURO.0487-23.2025.f4-2 Figure 4-2 Statistical summary of the GLM of the force data for Experiment 2 during the placement phase. (A-C) Statistical results table for force data related to coarse collection during the placement phase. Refer to (A-C) in Extended Data Figure 4-1 for visualized data. (D-F) Statistical results table for force data related to precise delivery during the placement phase. Refer to (D-F) in Extended Data Figure 4-1 for visualized data. (G-H) Statistical results table for transport and peg-free hand movement durations during the placement phase. Refer to (G-H) in Extended Data Figure 4-1 for visualized data. Download Figure 4-2, TIF file.

10.1523/ENEURO.0487-23.2025.f4-3 Figure 4-3 Evaluation of potential learning effects on force duration and GISI at peak force during the placement phase in Experiment 2. Traces indicate average force duration and GISI at peak force as a function of series with the anesthetized (red) and unanesthetized (blue) hands during each action for Block 2 (sham) and Block 3 (anesthesia). Tables indicate significant and non-significant slopes from linear model fits from all blocks (B1, B2, B3). Download Figure 4-3, TIF file.

Peg-free hand movement in the placement phase also exhibited changes in eye–hand coordination after anesthesia (Fig. 4*D*). With intact sensation, the gaze and hand positions were characterized by an initially large separation that decreased as they converged on the collection tray. In contrast, the gaze and hand positions remained in closer proximity throughout the movement action after the treated hand was anesthetized. A model accounted for this pattern with a significant block * hand * progress interaction ( $X_{8}^{2}$ = 254.8; *p* < 10^−16^). Post hoc tests revealed that the differences in gaze–hand separation measured for the treated hand after anesthesia compared with sham were significantly greater than the corresponding ΔGISI values for the untreated hand during all quartiles (onset, ΔΔGISI = −46.2 ± 4.5 mm; *z* = −10.4; *p* < 0.0001; AP_25_, ΔΔGISI = −53.0 ± 4.5 mm; *z* = −11.9; *p* < 0.0001; AP_50_, ΔΔGISI = −32.3 ± 4.5 mm; *z* = −7.3; *p* < 0.0001; AP_75_, ΔΔGISI = −25.3 ± 4.5 mm; *z* = −5.7; *p* < 0.0001; offset, ΔΔGISI = −25.3 ± 4.5 mm; *z* = −5.7; *p* < 0.0001). Similarly, differences in gaze–hand separation measured for the treated hand after anesthesia compared with baseline were significantly greater than the corresponding ΔGISI values for the untreated hand during all quartiles (onset, ΔΔGISI = −56.9 ± 4.5 mm; *z* = −12.8; *p* < 0.0001; AP_25_, ΔΔGISI = −73.3 ± 4.5 mm; *z* = −16.5; *p* < 0.0001; AP_50_, ΔΔGISI = −44.6 ± 4.5 mm; *z* = −10.0; *p* < 0.0001). Importantly, ΔGISI did not differ between the treated and untreated hands comparing sham against the baseline times (ΔΔGISI = 1.9 ± 2.0 mm; *z* = 0.9; *p* = 0.62). These results are consistent with the peg-free hand movement results in Experiment 1.

Following coarse collection, the pegs are transported and delivered to the pegboard. The separation between the gaze and hand at the time of peak force application during peg delivery (Fig. 4*E*) significantly differed between the treated and untreated hand (ΔΔGISI = −9.01 ± 3.5 mm, *z* = −2.6; *p* = 0.03) when comparing B1 and B3 (block * hand interaction; $X_{2}^{2}$ = 7.9; *p* = 0.03). With intact sensation, gaze and hand positions remain in close proximity during the initial course of precise delivery before gaze is released; however, GISI values for the anesthetized hand remain low for a more extensive portion of the action. These patterns (Fig. 4*F*) are captured by a significant a block * hand interaction ( $X_{2}^{2}$ = 123.0; *p* < 10^−16^) and a block * hand * progress interaction ( $X_{8}^{2}$ = 182.3; *p* < 10^−16^). Post hoc tests indicated that ΔGISI in the early AP was statistically indistinguishable between the treated and untreated hand. Importantly, post hoc across-hand comparisons revealed significant differences during the end of the precise delivery action comparing anesthesia to sham (B3–B2; offset, ΔΔGISI = −38.4 ± 3.8 mm; *z* = −10.263; *p* < 0.0001) and comparing anesthesia to baseline (B3–B1; offset, ΔΔGISI = −51.3 ± 3.75 mm; *z* = −13.8; *p* < 0.0001). These anesthesia effects occurred despite subtle sham effects (B2–B1; ΔΔGISI = 5.1 ± 1.7 mm; *z* = 3.0; *p* = 0.007) which were associated with gaze–hand separation increases. The anesthesia results are consistent with the precise placement results in Experiment 1.

Lastly, we evaluated gaze–hand relationships during peg transport (Fig. 4*G*). With intact sensation, the separation between gaze and hand positions was high at the beginning of transport, presumably reflecting a tendency for participants to look at the pegboard in advance of peg transport and the eventual convergence of gaze and hand positions. In contrast, gaze–hand separation was lower and relatively constant with the anesthetized hand. The model accounted for these patterns with a significant block * hand * progress interaction ( $X_{8}^{2}$ = 1,089.6; *p* < 10^−16^). Post hoc tests revealed significant ΔGISI differences between the treated and untreated hands during the initial three progress portions contrasting anesthesia and sham (B3–B2; onset, ΔΔGISI = −95.1 ± 3.4 mm; *z* = −27.7; *p* < 0.0001; AP_25_, ΔΔGISI = −94.9 ± 3.4 mm; *z* = −27.6; *p* < 0.0001; AP_50_, ΔΔGISI = −38.0 ± 3.4 mm; *z* = −11.0; *p* < 0.0001) and anesthesia and baseline (B3–B1; onset, ΔΔGISI = −100.0 ± 3.4 mm; *z* = −29.1; *p* < 0.0001; AP_25_, ΔΔGISI = −91.7 ± 3.4 mm; *z* = −26.7; *p* < 0.0001; AP_50_, ΔΔGISI = −33.1 ± 3.4 mm; *z* = −9.6; *p* < 0.0001). No hand differences were observed contrasting sham and baseline (B2–B1; ΔΔGISI = 1.5 ± 1.5 mm; *z* = 0.9; *p* = 0.61). These results are consistent with the peg transport results in Experiment 1.

### Experiment 2: eye–hand coordination in the retrieval phase is altered by digital anesthesia

**Figure 5.**
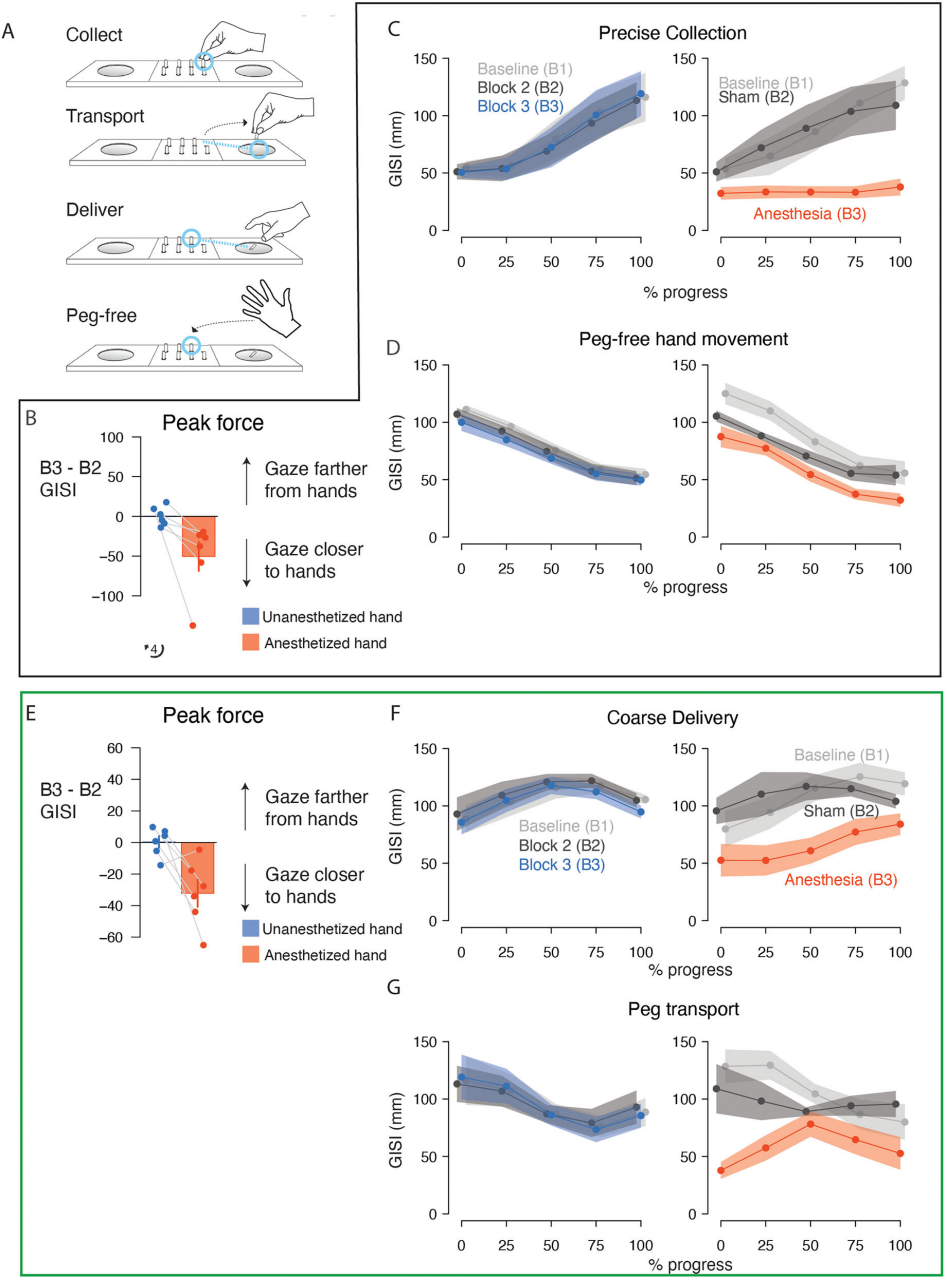
Experiment 2: gaze more closely tracked the hands in the retrieval phase following anesthesia treatment. ***A***, Schematic of the task during the retrieval phase in Experiment 2 divided into the constitutive actions. ***B***, Bar plots showing the mean change in GISI at peak force during precise collection between Block 2 (sham) and Block 3 (postanesthesia) with the nondominant (blue) and dominant (red) hands. The error bar indicate SEM. Connected markers indicate data from individual participants. ***C***, Lines and markers indicate mean GISI (average of participant-level means) calculated as a function of progress (%) through the precise collection action with the nondominant hand (left plot) and dominant hand (right plot). The shaded region indicates SEM. Light gray data indicate GISI in the baseline blocks. Dark gray data indicate GISI in Block 2 for the nondominant hand and dominant hand (sham). Blue data indicate GISI for the nondominant hand in Block 3. Red data indicate GISI for the dominant (anesthetized) hand in Block 3. ***D***, Lines and markers indicate mean GISI calculated as a function of progress (%) through the peg-free hand movement action. Conventions as in ***C***. ***E***, Bar plots showing the mean change in GISI at peak force during coarse delivery. Conventions as in ***B***. ***F***, Lines and markers indicate mean GISI calculated as a function of progress (%) through the coarse delivery action. Conventions as in ***C***. ***G***, Lines and markers indicate mean GISI calculated as a function of progress (%) through the peg transport action. Conventions as in ***C***. Force data for the retrieval phase of Experiment 2 is shown in Extended Data Figure 5-1. Summary statistics are provided in Extended Data Figure 5-2. Statistics of potential learning effects are provided in Extended Data Figure 5-3.

To examine how anesthesia influenced gaze–hand coordination while accounting for hand dominance and sham effects, we additionally analyzed data collected during peg-free hand movement, precise collection, transport, and coarse delivery in the retrieval phase (Fig. 5*A*).

10.1523/ENEURO.0487-23.2025.f5-1 Figure 5-1 Force results for Experiment 2 during the retrieval phase. Bar graphs of force data during the retrieval phase, i.e., peg pick-up from tray (coarse collection) and delivery to peg-holes (precise delivery) of experiment 2. (A-C) From coarse collection, bar graphs across all participants and conditions of duration of force application in the collection tray, peak normal force produced in the collection tray, and total torque force in the collection tray. (D-F) From precise delivery, bar graphs across all participants and conditions of duration of force application in the peg-hole, peak normal force produced in the peg-hole, and total torque force in the peg-hole. (G-H) Bar graphs depicting, for all trials sorted by condition, time elapsed between peg transport (completion of peg collection and initiating peg delivery for all trials sorted by condition), and peg-free hand movement (completion of peg delivery and initiating collection of the next peg). Bar graphs represent the mean with individual data points representing individual subjects. Error bar represents s.e.m. Download Figure 5-1, TIF file.

10.1523/ENEURO.0487-23.2025.f5-2 Figure 5-2 Statistical summary of the GLM of the force data for Experiment 2 during the retrieval phase. (A-C) Statistical results table for force data related to precise collection during the retrieval phase. Refer to (A-C) in Extended Data Figure 5-1 for visualized data. (D-F) Statistical results table for force data related to coarse delivery during the retrieval phase. Refer to (D-F) in Extended Data Figure 5-1 for visualized data. (G-H) Statistical results table for transport and peg-free hand movement durations during the retrieval phase. Refer to (G-H) in Extended Data Figure 5-1 for visualized data. Download Figure 5-2, TIF file.

10.1523/ENEURO.0487-23.2025.f5-3 Figure 5-3 Evaluation of potential learning effects on force duration and GISI at peak force during the retrieval phase in Experiment 2. Traces indicate average force duration and GISI at peak force as a function of series with the anesthetized (red) and unanesthetized (blue) hands during each action for Block 2 (sham) and Block 3 (anesthesia). Tables indicate significant and non-significant slopes from linear model fits from all blocks (B1, B2, B3). Download Figure 5-3, TIF file.

At peak force application during precise collection, we found that the gaze–hand separation for the treated and untreated hands changed differentially between the B2 (sham) and B3 (anesthesia) blocks (Fig. 5*B*; block * hand interaction; $X_{2}^{2}$ = 128.4; *p* < 10^−16^). A post hoc test confirmed that the change in GISI (sham vs anesthesia) for the treated hand differed significantly from the change in GISI for the untreated hand over the corresponding blocks (ΔΔGISI = −50.79 ± 4.75 mm; *z* = −10.7; *p* < 0.0001). The effect of anesthesia was also evident over the entire collection action (Fig. 5*C*); GISI steadily increased in the baseline and control conditions, while it remained low in the anesthesia condition. These patterns were captured by a significant block * hand * progress interaction ( $X_{8}^{2}$ = 232.8; *p* < 10^−16^). The across-hand differences in ΔGISI was significant in all action portions comparing B3 to B2 (onset, ΔΔGISI = −23.0 ± 5.2 mm; *z* = −4.5; *p* < 0.0001; AP_25_, ΔΔGISI = −44.5 ± 5.2 mm; *z* = −8.6; *p* < 0.0001; AP_50_, ΔΔGISI = −71.7 ± 5.2 mm; *z* = −13.9; *p* < 0.0001; AP_75_, ΔΔGISI = −95.4 ± 5.2 mm; *z* = −18.5; *p* < 0.0001; offset, ΔΔGISI = −95.2 ± 5.2 mm; *z* = −18.5; *p* < 0.0001) and B3 to B1 (onset, ΔΔGISI = −20.3 ± 5.2 mm; *z* = −3.9; *p* = 0.0003; AP_25_, ΔΔGISI = −33.7 ± 5.2 mm; *z* = −6.5; *p* < 0.0001; AP_50_, ΔΔGISI = −54.9 ± 5.2 mm; *z* = −10.6; *p* < 0.0001; AP_75_, ΔΔGISI = −85.8 ± 5.2 mm; *z* = −16.6; *p* < 0.0001; offset, ΔΔGISI = −103.6 ± 5.2 mm; *z* = −20.1; *p* < 0.0001). In contrast, a significant block * hand interaction ( $X_{2}^{2}$ = 996.0; *p* < 10^−16^) and subsequent post hoc analyses revealed significant across-hand differences in ΔGISI comparing sham and baseline conditions (B2–B1; ΔΔGISI_B2_–_B1_ = 6.3 ± 2.3 mm; *z* = 2.7; *p* = 0.02), indicating that gaze–hand separation increased slightly following sham injection. These anesthesia results are consistent with the precise collection results in Experiment 1.

In all conditions, the peg-free hand movement action before precise collection was characterized by a monotonic reduction in the separation between gaze and hand positions (Fig. 5*D*). While there was substantial overlap in the GISI traces for the untreated hand over B1, B2, and B3, the GISI traces for the treated hand exhibited differences with larger values in the sham condition and smaller values in the anesthesia condition. These patterns were captured by a significant block * hand interaction ( $X_{2}^{2}$ = 207.7; *p* = 10^−16^). The block * hand * progress interaction achieved significance ( $X_{8}^{2}$ = 15.5; *p* = 0.05) despite the similar dynamics in all conditions. Post hoc tests revealed significant ΔGISI differences between the treated and untreated hands contrasting B3 to B2 (onset, ΔΔGISI = −16.5 ± 3.4 mm; *z* = −4.2; *p* = 0.0001; AP_50_, ΔΔGISI = −15.2 ± 3.4 mm; *z* = −3.9; *p* = 0.0003; AP_75_, ΔΔGISI = −21.3 ± 3.4 mm; *z* = −5.4; *p* < 0.0001; offset, ΔΔGISI = −25.0 ± 5.2 mm; *z* = −6.3; *p* < 0.0001) and to B1 (onset, ΔΔGISI = −28.7 ± 3.4 mm; *z* = −7.3; *p* < 0.0001; AP_25_, ΔΔGISI = −22.4 ± 3.4 mm; *z* = −5.7; *p* < 0.0001; AP_50_, ΔΔGISI = −24.2 ± 3.4 mm; *z* = −6.2; *p* < 0.0001; AP_75_, ΔΔGISI = −25.3 ± 3.4 mm; *z* = −6.4; *p* < 0.0001; offset, ΔΔGISI = −22.8 ± 3.4 mm; *z* = −5.8; *p* < 0.0001). Importantly, across-hand ΔGISI differences were also significant comparing B3 to B2 (ΔΔGISI = −17.26 ± 1.8 mm; *z* = −9.8; *p* < 0.0001), so the loss of sensory feedback with the dominant hand resulted in an even tighter association between spatial association between gaze and hand positions compared with that achieved with the sham injection, which were also significant (ΔΔGISI = −7.4 ± 1.8 mm; *z* = −4.2; *p* < 0.0001). These results are consistent with the peg-free action results in Experiment 1.

Gaze–hand relationships differed significantly at the time of peak force production during coarse delivery (block * hand interaction; $X_{2}^{2}$ = 69.3; *p* = 10^−16^; Fig. 5*E*). Post hoc tests revealed significant differences between ΔGISI contrasting B3 and B2 for the treated and untreated hand (ΔΔGISI = −32.6 ± 4.5 mm; *z* = −7.2; *p* < 0.0001). A tighter spatial relationship between the gaze and anesthetized hand was also evident throughout coarse delivery action in comparison with the other conditions (Fig. 5*F*). A model accounted for these patterns with a significant block * hand * progress interaction ( $X_{8}^{2}$ = 88.3; *p* = 10^−15^). Post hoc tests indicated significant across-hand differences throughout the coarse delivery action in ΔGISI comparisons between B3 and B2 (onset, ΔΔGISI = −46.9 ± 5.0 mm; *z* = −9.4; *p* < 0.0001; AP_25_, ΔΔGISI = −56.2 ± 5.0 mm; *z* = −11.3; *p* < 0.0001; AP_50_, ΔΔGISI = −55.8 ± 5.0 mm; *z* = −11.2; *p* < 0.0001; AP_75_, ΔΔGISI = −32.7 ± 5.0 mm; *z* = −6.6; *p* < 0.0001; offset, ΔΔGISI = −14.2 ± 5.0 mm; *z* = −2.9; *p* = 0.01) and between B3 and B1 (onset, ΔΔGISI = −22.8 ± 5.0 mm; *z* = −4.6; *p* < 0.0001; AP_25_, ΔΔGISI = −42.2 ± 5.0 mm; *z* = −8.5; *p* < 0.0001; AP_50_, ΔΔGISI = −60.4 ± 5.0 mm; *z* = −12.1; *p* < 0.0001; AP_75_, ΔΔGISI = −45.1 ± 5.0 mm; *z* = −9.1; *p* < 0.0001; offset, ΔΔGISI = −25.4 ± 5.0 mm; *z* = −5.1; *p* < 0.0001). No significant hand differences were observed comparing B2 and B1 (ΔΔGISI = 2.0 ± 2.2 mm; *z* = 0.9; *p* = 0.64). These results are consistent with the coarse delivery results in Experiment 1.

Lastly, we evaluated eye–hand coordination during peg transport leading up to coarse delivery. A significant block * hand * progress interaction ( $X_{8}^{2}$ = 312.8; *p* = 10^−16^) captured the GISI dynamics which were characterized by smaller separations between gaze and the treated hand in the anesthesia block (Fig. 5*G*). Across-hand comparisons revealed significant differences in ΔGISI contrasting B3 and B2 at all AP points (onset, ΔΔGISI = −95.2 ± 4.8 mm; *z* = −19.7; *p* < 0.0001; AP_25_, ΔΔGISI = −54.4 ± 4.8 mm; *z* = −11.3; *p* < 0.0001; AP_50_, ΔΔGISI = −18.3 ± 4.8 mm; *z* = −3.8; *p* = 0.0004; AP_75_, ΔΔGISI = −32.9 ± 4.8 mm; *z* = −6.8; *p* < 0.0001; offset, ΔΔGISI = −46.9 ± 4.8 mm; *z* = −9.7; *p* < 0.0001). Notably, the across-hand ΔGISI comparison comparing B2 to B1 failed achieved significance (ΔΔGISI = 0.4 ± 2.2 mm; *z* = 0.2; *p* = 0.98), indicating that sham injection did not affect the spatial relationship between the gaze and treated hand. More critically, the effect of anesthesia on gaze–hand relationships is consistent with the peg transport effects in Experiment 1.

## Discussion

In this study, we examined eye–hand coordination during a dexterous motor task when fingertip somatosensation was acutely abolished. In two experiments, participants performed a unimanual pegboard task before and after digital anesthesia was administered to the thumb, index finger, and middle finger on one of the hands. We separated the task into placement and retrieval phases, each consisting of four specific actions: peg-free hand movement, peg collection, transport, and delivery. Because pegs were collected from and delivered to large and small holding containers, we examined whether somatosensory feedback loss impacted task performance in a manner that depended on specific actions according to their required precision. In both experiments, participants continued to complete the task successfully with insensate fingers, albeit with longer trial times and altered force profiles. When examining the gaze–hand spatial relationship throughout the task, we found that gaze was generally directed closer to the hand under anesthesia. Compensatory changes in gaze behavior reflected an adaptive strategy to maintain task performance by increasing visual monitoring of the anesthetized hand. This compensation occurred when the dominant or nondominant hand was anesthetized and were significantly greater than the subtle changes observed in the sham injections. Because compensatory changes were evident in all task actions, even when no peg was grasped, we conclude that the acute removal of tactile feedback was associated with nonspecific modulation of eye–hand coordination.

We first determined whether digital anesthesia impacted performance on the pegboard task. Timing and force data revealed the importance of cutaneous responses to task performance (Extended Data Figs. 2-1, 2-2, 3-1, 3-2, 4-1, 4-2, 5-1, 5-2), consistent with earlier work (Augurelle et al., 2003; Monzée et al., 2003; Sober and Sabes, 2005; Trillos et al., 2018). First, participants took more time to complete collection and delivery following anesthesia, regardless of the precision required. The increased time required to complete the actions successfully could be due to altered manual dexterity resulting from the loss of cutaneous touch. Alternatively, but not exclusively, the increased time could reflect participants' switch to relying on visual feedback with the reasonable assumption that dexterous actions are slower when supported by visual feedback as compared with somatosensory feedback. Second, participants produced greater normal forces on the pegboard during all collection events and greater torque forces during precise collection events. Additionally, participants produced greater torque forces during precise delivery events. These results imply that somatosensory feedback is critical for force regulation. Lastly, peg transport times and peg-free hand movement were longer following anesthesia. These timing results suggest general adaptive changes that are not linked to specific task phases or even grasping, consistent with previous findings (Gentilucci et al., 1997). Collectively, these findings confirm that digital anesthesia impacted participants' ability to perform the pegboard task. Although participants were able to complete the task, the duration and force changes indicate that adaptations following the loss of somatosensory function only provided a partial rescue of function.

Our main finding was that digital anesthesia resulted in reduced spatial separation between gaze and hand position. This change in eye–hand coordination behavior was evident in all task actions. Following anesthesia, a smaller distance separated gaze and hand positions during collection and delivery actions, which suggests that participants relied more on visual feedback for peg interactions following the loss of somatosensory signals. The most obvious effect was seen with coarse collection where gaze is consistently close to the hand throughout the collection epoch in the anesthetized condition. In contrast, there was a greater gaze–hand separation in all other conditions that increases over the coarse collection epoch as participants looked away from the pegs and toward the upcoming placement targets. Gaze changes were more subtle with precise actions, which likely reflect a greater baseline demand for visual support of precise actions even with intact somatosensation. Indeed, gaze was closely tied to hand position during precise delivery and collection in all conditions, and anesthesia-related gaze changes were only evident at the end of the action. This pattern reflects vision's role in providing feedback regarding the success of peg collection or placement. However, with intact tactile sensation, vision was more quickly released from this feedback function, presumably to guide the upcoming hand movement (Blouin et al., 1996). This pattern is consistent with previous reports that different phases of prehension and movement can be performed without the necessity of visual feedback (Gentilucci et al., 1994). Notably, after digital anesthesia, we found that participants gazed longer at their hands through the actions rather than disengaging to support guidance for the upcoming movement. These collective results support a conceptual framework in which vision and somatosensation serve as action-phase controllers during dexterous manipulation tasks that require eye–hand coordination (Johansson et al., 2001) and imply that vision becomes essential for achieving task subgoals when tactile “checkpoints” are unreliable.

We found that participants continued to look closer to their hands during movement actions of the task following digital anesthesia. This effect was clearest with peg transport toward precise placement. Prior to anesthesia in the control conditions, gaze and hand behaviors appear to be independent. Accordingly, the transport action was characterized by a large initial separation between gaze and hand positions as participants looked toward the pegboard targets while independently completing peg collection. The gaze–hand separation gap then converged over the transport epoch as the peg was transported to the pegboard target where gaze was fixated. These results are consistent with previous studies suggesting that afferent information contributes to the adjustment between motor command and visual information (Vercher et al., 1996; Stenneken et al., 2006). We observed a different pattern with anesthesia: gaze remained closely tied to the hand throughout the transport action. This partially reflects the gaze–hand relationship during the preceding (collection) action, but it also reveals that participants looked closer to their hands as they transported the pegs, conceivably to monitor whether grasp was successfully maintained. We observed a similar pattern during transport toward coarse delivery, producing a behavior consistent with the hypothesis that vision is recruited to provide feedback when somatosensation is perturbed. Moreover, we found that during anesthesia, participants looked closer to their hands and needed more time in movement actions even when they were not grasping a peg. This surprising result implies that gaze behavior changes are not specifically tied to actions involving object interactions but also to the preparatory phase before object contact. Our study investigated the loss of sensory information on eye–hand coordination, and together our data support the notion that adaptation of eye–hand coordination following acute somatosensory feedback loss may be applied broadly rather than to specific actions.

We considered that the effects of digital anesthesia on task performance and compensatory behaviors may have depended on hand dominance. Conceivably, anesthesia effects could have been more pronounced with the nondominant hand assuming this hand is less dexterous. To address this possibility, we focused on the nondominant hand in Experiment 1 and the dominant hand in Experiment 2. In both experiments, performance with the dominant and nondominant hands was comparable in the baseline blocks. More importantly, we found that anesthesia disrupted performance and induced gaze–hand changes in both experiments which indicates that the typical increased reliance on the dominant hand did not protect against the deleterious impacts of acute anesthesia.

In Experiment 2, we additionally tested the dominant hand after the participants received a sham injection. This manipulation allowed us to determine whether the mere experience of receiving an injection – with sensation left intact or even heightened – could have altered the salience of the hand and participants' use of the hand. Across task phases, gaze behavior following sham injection mostly matched baseline behavior, although there were subtle differences in some actions. Notably, the gaze–hand separation in some actions “increased” following sham injection compared with the baseline, suggesting that participants relied “less” on their gaze as they gained experience on the task. Indeed, we found a significant block * hand interaction in the collect and delivery actions in the placement phase and the collection action in the retrieval phase, which is consistent with learning effects within the blocks (Wolpert and Flanagan, 2010). Motivated by these observations, we performed exploratory analyses to quantify potential learning effects. We tested whether the duration of force application and GISI at peak force changed within each block for each hand separately (Extended Data Figs. 2-3, 3-3, 4-3, 5-3). Although we found significant linear trends in some conditions, these effects were inconsistent across experiments and tended to be small. Some trends followed learning effect predictions (i.e., shorter force durations and increased GISI over series); however, trends in the opposite direction were also observed. Crucially, any changes in force duration or GISI associated with potential learning effects were substantially smaller than digital anesthesia effects. The effects of sham and anesthesia matched in direction (i.e., reduced gaze–hand separation) only during the peg-free movements of the retrieval phase (Fig. 5*D*, right), but the significant sham effects may have been related to the disproportionately large gaze–hand separations with the dominant hand in the baseline block. In all analyses, the influence of anesthesia on gaze–hand relationships dwarfed those seen with sham injection or learning.­

While the effects shown in this study are clear, further experimental modifications can improve the paradigm and expand the findings. First, we have a limited sample size with gaze data from 10 participants across two experimental groups. However, by testing each participant extensively, with 1,280 trials per participant in Experiment 1 and 1,920 trials per participant in Experiment 2, we systematically determined each participant's performance with and without digital anesthesia with robustness. Our data revealed robust effects of anesthesia that replicated over two independent experiments. Although there were subtle individual differences that likely reflected the strategies employed to complete the task, we leveraged this variance in our models which included trial-level data from the experimental and control hands of each subject. Despite confidence in the results of our small-sampled experiments, the generalizability of our conclusions must be evaluated in future studies with larger samples. Next, in applying digital anesthesia to the fingers, the positional sense of fingers may have been affected in addition to the cutaneous sensibilities. Because of the importance of proprioception to prehension and grasping (Gentilucci et al., 1997), the sensitivity of cutaneous mechanoreceptors to hand kinematics (Edin and Abbs, 1991), and modulatory influence of cutaneous vibrations on proprioceptive acuity (Weerakkody et al., 2007), perturbed proprioception may have contributed to the disrupted performance on our task. We neglected to evaluate position sense with the anesthetized fingers, so the role of proprioception (loss) in gaze–hand adaptation remains to be tested. Furthermore, we did not track the nonanesthetized fingers, and participants may have shifted their gaze to these fingers. We think this is unlikely for multiple reasons. First, due to the natural hand orientation during the task, these fingers were partially occluded by the radial digits. Second, continuous visual monitoring by the experimenter also confirmed that these fingers did not participant in grasping the pegs in any of the trials. However, without direct quantification of finger-specific gaze allocation or visibility, we cannot rule out the influence of neighboring finger movement. Third, the lack of consistent trial structure, particularly with respect to the peg order that participants needed to complete the delivery and collection actions, precluded us from careful analysis into the pattern of gaze behavior based on pegboard position. Future studies incorporating a systematic peg selection order may better assess the impact of visuomotor strategy on eye–hand coordination behavior and motor planning. Lastly, our analyses of gaze–hand relationships were handicapped by the limited sampling rate of our gaze tracking system. With a higher sampling rate, more sensitive dynamics in gaze–hand coordination may be observed.

In conclusion, we found that the loss of somatosensory feedback through digital anesthesia impairs performance on a manual dexterity task. Learning effects from previous experience with the task were lost following anesthesia, and behavioral adaptations only partially rescued performance. To compensate for the loss of touch, participants consistently directed their gaze closer to their hands, which we interpret as the recruitment of vision for feedback functions typically supported by touch. That gaze became more closely tied to the hands following anesthesia even in the absence of peg grasping implies that adaptations to dexterous behaviors may be more general in nature. Although our results reveal that gaze–hand behavior adaptations generalize across task phases in our paradigm, an open question is whether they would extend to other tasks. Future studies can address this question as well as whether our compound eye–hand coordination assessment technique can reveal adaptations in persons that suffer from chronic somatosensory feedback impairment. Similarly, this approach provides biomarkers of adaptive changes after peripheral nerve injury affecting sensation in the hand and offers a valuable tool for assessing the impact of clinical interventions aimed at restoring function. Clinicians can track these mechanisms to evaluate recovery after nerve repair and compare treatments. Such insights are crucial for translating sensorimotor research into clinical applications, ultimately enhancing outcomes for individuals with sensory deficits or motor impairments. Future work could determine whether the effect of learning persists after tactile feedback has returned, shedding light on the durability of sensorimotor adaptations. Finally, expanding our understanding of how dexterous behavior patterns change with altered somatosensory and visual feedback may also inform the design of neuroprosthetics and optimize training regimens for advanced robotic telesurgery.

## Synthesis

Reviewing Editor: David Franklin, Technische Universitat Munchen Decisions are customarily a result of the Reviewing Editor and the peer reviewers coming together and discussing their recommendations until a consensus is reached. When revisions are invited, a fact-based synthesis statement explaining their decision and outlining what is needed to prepare a revision will be listed below. The following reviewer(s) agreed to reveal their identity: Lucy Dowdall.

Your manuscript has been re-reviewed by the two original reviewers. They both find the work compelling and highlight the advance that this work potentially provides. While they both appreciate the revisions that you have made and the new experimental additions, they still have similar concerns regarding the current version of the manuscript - particularly the statistics and potential sample size issues along with several other issues. In addition, it appears that your link to the code and data has been excluded due to the double-blind procedure, which means that the reviewers cannot examine the material. Please consider a version which would allow this to be shared. Please use their concerns and comments which are detailed below as you revise your manuscript.

Reviewer 1

The authors thoroughly improved the manuscript, and I appreciate the testing of a new sample of participants.

There are, however, still some points that need clarification in the methods, analysis, and results.

Here are my comments:

In general, the method and the results are hard to follow. There is a lack of consistency in the language, and is very hard to understand the experimental design and the results. This is mainly complicated by the use of the words "series", "rounds" , and" trials" without clearly stating what they refer to. The task seems to nest different components: trials/series are nested into series/round, which are nested into rounds/block. This is very confusing, as the statistical model doesn't seem to consider all these nested factors.

I appreciate that the authors conducted a second experiment with a new sample of six participants. However, my previous concerns regarding the GLM remain, particularly now that the model includes the factor "Hand." In this context, the "Hand" factor effectively corresponds to the experiment itself: Experiment 1 involved anesthetizing the non-dominant hand, while Experiment 2 involved anesthetizing the dominant hand. As a result, the model continues to estimate interactions such as Block*Hand, Block*Phase, and Block*Hand*Phase using data from only five and six participants, respectively. This sample size is quite limited for the reliable estimation of such interactions. To strengthen the validity of the analysis, I recommend that the authors conduct a power analysis, taking into account the number of trials and the expected variability in the data. This would help determine the minimum sample size required to achieve robust and interpretable estimates.

In this context, the sample size for Experiment 2 should be increased, as it involves testing the dominant hand and currently includes only six participants. Conducting a power analysis would help determine the minimum number of participants needed, taking into account the experimental design, nested factors (e.g., block, round, series, trials), and expected variability. The data from these six participants can serve as pilot data to inform this analysis. The same approach should also be applied to the Experiment 1. A valuable reference for estimating sample size in the context of mixed models is DeBruine, L. M., &Barr, D. J. (2021). Understanding mixed-effects models through data simulation. Advances in Methods and Practices in Psychological Science, 4(1), 2515245920965119. One suggestion is for the authors to simulate sample size requirements using a bootstrap approach. This could involve utilizing the correlation matrices and observed variability from the current samples (five participants in Experiment 1 and six in Experiment 2, separately), while keeping the number of trials constant and varying the number of participants (e.g., from 5 to 20). For each sample size, the simulation should be repeated 10,000 times to extract F-values for each main effect and interaction. A similar methodology is described by Vasishth, S., Mertzen, D., Jäger, L. A., &Gelman, A. (2018). The statistical significance filter leads to overoptimistic expectations of replicability. Journal of Memory and Language, 103, 151-175, in the context of Bayesian hierarchical models. While their approach is Bayesian, the simulation and modeling framework is analogous and can serve as a useful reference for applying similar techniques to mixed-effects models.

It is not clear how the authors controlled and measured the level of attention in the participants for Experiment 2.

Additionally, I found the following sentence (Page 14, line 335) ambiguous: "From all models, we simplified the fixed effects, random effects, or both to fit the most comprehensive model that still converged." It is unclear what is meant by "simplified." Typically, model selection is guided by balancing goodness-of-fit and parsimony, often using criteria such as AIC or BIC to avoid overfitting. The authors do not report such comparisons, and the inclusion of additional complexity (e.g., Phase and Hand) may result in a model that is unnecessarily complicated given the small sample size. This is especially relevant in light of the three-way interaction the authors found in their results.

A further concern relates to the factor "Block," whose levels differ across experiments. As stated on Page 13, line 317: "we included block (Experiment 1: baseline vs post-anesthesia; Experiment 2: baseline vs sham vs anesthesia)" This implies that the model is estimating a three-way interaction using a factor "Block" that has a different levels in each experiment: experiment 1 contains two sublevels (baseline vs post-anesthesia) and experiment 2 with 3 sublevels (baseline vs sham vs anesthesia). In such cases, it would be more appropriate to treat Experiment (or "Hand," as currently defined in the manuscript) as a random effect to reflect the hierarchical structure of the data. However, this would lead to a further problematic issue related to the low number of participants in each experiment.

The explanation of "series" and "rounds" is unclear. It is not evident what distinguishes a "series" from a "round," and why the authors chose to model Series using the random effect structure (0 | Subject/Series). This implies that each subject has a unique series, which does not seem to be the case. The pegs were positioned in the same way for each subject and in the same position. If each subject performed the same set of peg movements (i.e., no variability in the items or task structure), a more suitable approach to analyzing learning effects would be to either:

1) Model trial number as a fixed effect to assess learning trends over time (e.g., by slope), or

2) Bin trials into meaningful chunks and include them as a categorical factor to capture non-linear learning trajectories.

As currently implemented, the model structure appears misaligned with the experimental design, potentially undermining the interpretability of the findings.

The learning effect is particularly relevant given the findings in Dempsey-Jones, H., Themistocleous, A. C., Carone, D., Ng, T. W. C., Harrar, V., &Makin, T. R. (2019). Blocking tactile input to one finger using anaesthetic enhances touch perception and learning in other fingers.Journal of Experimental Psychology: General, 148(4), 713-727. https://doi.org/10.1037/xge0000514. There is no mention of the movement of neighbouring fingers in peg placement. It could be that eye movements were synchronized to the movements of the intact fingers rather than the anesthetized ones.

Regarding the peg position, it is possible that eye-hand coordination changes according to the order in which the pegs are picked up and placed. It may be that those who start with the peg closest to the hand start position have a different strategy than those who start with the peg positioned far from the hand start position. Were there any instructions given to the participants regarding the first pegs to pick up, in this case?

The description of the procedure and methods is quite confusing. On page 14, line 348, the authors state:"The task was split into two phases: placement or retrieval of pegs. Within each phase, we defined 4 actions using the force signals measured at the collection trays and pegboard: peg-free hand movement, peg collection, peg transport, and peg delivery." Figure 4 is meant to represent these four actions, with each action depicted as a signal ranging from 0 to 100%. However, on page 8, line 196, the authors write: "For each trial, we identified the timing of collection, transport, and delivery events based on the force variations measured on the collection and peg-board trays." This creates confusion. Figure 4 suggests that each of the 4 "actions" unfolds continuously over time, represented as a full signal spanning from 0% to 100% of the movement duration. In contrast, the description on page 8 implies that these actions are discrete events that occur at specific time points along the hand trajectory. Given this, I expected to see clear time-stamped data for each distinct event (peg-free movement, collection, transport, and delivery) rather than continuous signals. The inconsistency between the figure and the textual description makes the methodology unclear and difficult to interpret.

Minor comments

It would have been useful to have the changes tracked by evidencing them in a color other than black. This would have eased the comparison with a previous version of the manuscript.

Page 5 line 105: it may be worth repeating that Experiment 1 was related to the non-dominant hand, and Experiment 2 to the dominant hand by stating it within parentheses, for instance: (Experiment 1 - non-dominant hand)

Page 12 line 281:" This distinction allowed us to test the hypothesis that gaze-hand position relationships depend on the task difficulty" The task difficulty, however, was not modeled in the mixed model. This is a bit confusing.

Reviewer 2

The paper benefits from the redirection of focus to the gaze-hand coordination, offering unique insights into compensatory behaviours when somatosensory feedback is abolished. The manuscript is very well written, particularly notable in the excellent review of the literature in the introduction that elegantly leads to the study aims, in addition to the comprehensive discussion. I appreciate the extensive efforts of the authors to improve on the experimental design following review in December 2023, and was particularly impressed with additional controls utilised in experiment two. My previous comments were all carefully responded to. The methodology is sound and the results section thoroughly covers the visual feedback compensation used during various types of movement, including both precise and coarse movements. The manuscript is in very good shape, I just have a few concerns to be addressed.

Major Comments

1. I understand the use of trial-level data to allow complex modelling with a limited sample size. However, I do still have remaining concerns about just how small the sample is vs the complexity of the models fitted. Estimates of the between-subjects variability is likely underpowered. Relatedly, the statement in the discussion "Our data revealed high consistency across participants within each experiment" does not seem to have been demonstrated by the results provided. More caution should be taken in the discussion regarding the limits of generalisability based on the small sample.

2. Additionally, I did not receive access to the "code included as Extended Data" that should have included the final models for each analysis. Could the authors please provide the final models for each? Was it always ensured that the repeated-measures structure of the data was appropriately accounted for?

3. I could not locate the statistical analysis for the time taken and force production, that led to the statement "digital anesthesia resulted in significant changes in the time required to complete the actions and the forces produced by the actions" (line 359-360). Could the authors please ensure this analysis is included somewhere - I think the manuscript would benefitting from including this result in the main text, instead of just being included in the extended data.

4. Each set of post-hoc tests following a significant interaction should be corrected for multiple comparisons.

5. Did the authors observe any correlations between GISI changes and time taken or force modulation in the anaesthetised hand?

Minor Comments

Methods

Were participants specifically instructed to use a precision grip with their Thumb and index/middle, or given that it was strategy-free, would it have been allowed to compensate by using a non-anaesthetised finger, e.g. Thumb with the ring finger? If so, were such compensatory behaviours observed?

Were participants blinded to when they were receiving the sham injection vs the anaesthetic, or were they told the ingredients of each set of injections before they were administered?

Was the 2PD threshold always exceeding 15mm after the anaesthesia (consistent across every single participant)? I would like to see the thresholds visualised as much as possible with the data available, including at baseline vs after the sham injections. Additionally, was the participant's 2PD threshold re-assessed following the final anesthetised block, to assess maintenance of the deafferentation?

Once no force is being applied to the force transducers, how long does it take the normal force to return to baseline levels (the latency)?

Were there any issues with the hand going out of the tracking frame, and if so, how was this accounted for?

Results

The paragraph beginning on line 367 discuss the temporal offset metrics. The paragraph includes results from the "test series" and "retrieval phase" , that appear to be separate time periods within the trial. However, series is earlier defined as the 8 placement and 8 retrieval trials. It is therefore not clear what the difference is in the test series vs the retrieval phase is. Does the test series include everything, or only the placement?

The interaction on line 525 does not have any follow-up tests to confirm direction?

In Figures 2-5, a legend or label for the blue and red bars for the Peak Force would help readability

In the Extended Data Figures, the legend could be improved for support the reader by stating which colours correspond to which condition (e.g. blue for control, red for anesthetised hand), rather than just 'time 1', 'time 2'.

## Author Response

We appreciate the careful review and feedback provided by the reviewers regarding our manuscript, "Acute loss of tactile input leads to general compensatory changes in eye-hand coordination during object manipulation" (eN-NWR-0487-32). Both reviewers found the work compelling and appreciated our responses to the first round of reviews, including our replication study. Each reviewer provided additional comments and questions. Both reviewers noted a concern about statistical power given the sample sizes of our 2 experiments. To alleviate this concern, we performed power analyses according Reviewer 1's recommendations. We describe the power analyses, analyses related to potential learning effects, statistics from sensory testing, access to code/data, and responses to all other points raised by reviewers below. We address each point and describe corresponding revisions made to the manuscript.

Reviewer 1 Q1.1 In general, the method and the results are hard to follow. There is a lack of consistency in the language, and is very hard to understand the experimental design and the results. This is mainly complicated by the use of the words "series", "rounds" , and" trials" without clearly stating what they refer to. The task seems to nest different components: trials/series are nested into series/round, which are nested into rounds/block. This is very confusing, as the statistical model doesn't seem to consider all these nested factors.

We apologize for the confusion about the terminology. The reviewer is correct in interpreting the nested structure of the experiment. We have rewritten the methods to clarify the experimental design and revised the diagram in Figure 1: "Each experiment (Fig. 1D) comprised multiple blocks defined according to the state of the hand receiving the injections (Experiment 1: non-dominant hand; Experiment 2: dominant hand). Experiment 1 comprised 2 blocks: baseline (no intervention) and anesthesia. Experiment 2 comprised 3 blocks: baseline, sham injection, and anesthesia. Injections (see below) were performed immediately following block 1 in Experiment 1 and blocks 1 and 2 in Experiment 2. Basic sensory and motor capabilities (see below) were evaluated prior to each block and following the final block.

Each block consisted of testing of the dominant and non-dominant hands. Each hand was tested in 4 rounds consecutively. Participants were offered a short break (1-3 minutes) between rounds. Gaze- and hand position tracking calibration was performed prior to the start of each round.

Each round comprised 5 series. At the start of each series, the hands were placed on top of handrests equipped with light sensors. Series were divided into placement and retrieval phases. An acoustic signal indicated when participants should begin each phase. During the placement phase, participants moved the pegs, one at a time, from the tray to the peg holes. The collection and delivery of a single peg defined a placement trial. After placing all 8 pegs, participants returned the active hand to the handrest. After a 4-second pause, an acoustic signal prompted participants to begin the retrieval phase. During the retrieval phase, participants moved the pegs, one at a time, from the peg holes to the collection tray. The collection and return of a single peg defined a retrieval trial. After returning all the pegs to the collection tray, participants placed the active hand back on the handrest to signal the end of the series.

Participants were instructed to use a precision grip to grasp and place the pegs. No instructions were given regarding how to collect the pegs (other than one at a time), regarding the order of the pegs, or regarding gaze direction (i.e., the participants were free to find their own strategy). If a peg was dropped, participants were instructed to continue by picking up a new peg and leaving the dropped peg for last.

In Experiment 1, each participant performed 1280 total trials. In Experiment 2, each participant performed 1920 total trials." Q1.2 I appreciate that the authors conducted a second experiment with a new sample of six participants. However, my previous concerns regarding the GLM remain, particularly now that the model includes the factor "Hand." In this context, the "Hand" factor effectively corresponds to the experiment itself: Experiment 1 involved anesthetizing the non-dominant hand, while Experiment 2 involved anesthetizing the dominant hand. As a result, the model continues to estimate interactions such as Block*Hand, Block*Phase, and Block*Hand*Phase using data from only five and six participants, respectively. This sample size is quite limited for the reliable estimation of such interactions. To strengthen the validity of the analysis, I recommend that the authors conduct a power analysis, taking into account the number of trials and the expected variability in the data. This would help determine the minimum sample size required to achieve robust and interpretable estimates.

In this context, the sample size for Experiment 2 should be increased, as it involves testing the dominant hand and currently includes only six participants. Conducting a power analysis would help determine the minimum number of participants needed, taking into account the experimental design, nested factors (e.g., block, round, series, trials), and expected variability. The data from these six participants can serve as pilot data to inform this analysis. The same approach should also be applied to the Experiment 1. A valuable reference for estimating sample size in the context of mixed models is DeBruine, L. M., &Barr, D. J. (2021). Understanding mixed-effects models through data simulation. Advances in Methods and Practices in Psychological Science, 4(1), 2515245920965119. One suggestion is for the authors to simulate sample size requirements using a bootstrap approach. This could involve utilizing the correlation matrices and observed variability from the current samples (five participants in Experiment 1 and six in Experiment 2, separately), while keeping the number of trials constant and varying the number of participants (e.g., from 5 to 20). For each sample size, the simulation should be repeated 10,000 times to extract F-values for each main effect and interaction. A similar methodology is described by Vasishth, S., Mertzen, D., Jäger, L. A., &Gelman, A. (2018). The statistical significance filter leads to overoptimistic expectations of replicability. Journal of Memory and Language, 103, 151-175, in the context of Bayesian hierarchical models. While their approach is Bayesian, the simulation and modeling framework is analogous and can serve as a useful reference for applying similar techniques to mixed-effects models.

We want to clarify that the "Hand" factor in the models captures differences between the dominant and non-dominant hands which were tested in each participant in both experiments. Accordingly, including the "Hand" factor in Hand*Block interactions captures the effects of anesthesia in our models (our effect of interest) while accounting for the non-treated hand as an internal control.

Based on the reviewer's suggestion, we conducted a power analysis to determine the number of subjects required to detect our observed effects with sufficient power. Our approach followed the reviewer's exact prescription and we implemented power analyses using the SIMR package (Green &MacLeod, 2015; "SIMR: an R package for power analysis of generalized linear mixed models by simulation") testing the exact LME specifications in our reported analyses. Given that our primary conclusions center around results from 3 main analyses, we focused our power analyses on these results: 1) Anesthesia alters task performance as measured by the duration of force application, 2) Anesthesia is associated with a reduction in the separation between gaze and hand position when participants engage with the pegs as measured by GISI at peak force application, and 3) Anesthesia is associated with GISI reduction during hand movements with and without pegs. Explicitly, our power analyses determined how likely the null hypothesis can be rejected at different sample sizes given our observed effects size assuming the trial number and variability in our data. For each analysis, we simulated the experiments 1000 times for each sample size comparing a model with the critical interaction term to a model assuming no interaction. Below, we plot the power curves for the force and GISI effects during each action for Experiments 1 and 2 (24 total tests). In the analyses reported in the main text and supplement, significant effects were reported in 22/24 tests. Power analyses revealed that >99% power is achieved with 4 subjects for 20/22 significant effects we reported. For the remaining 2 reported significant results, >80% power is achieved with 4 subjects.

Q1.3 It is not clear how the authors controlled and measured the level of attention in the participants for Experiment 2.

We did not mean to imply that we controlled or measured participants attention. A reviewer had commented on our initial submission that the mere fact that one hand received an injection may have altered the use of that hand. This concern motivated our inclusion of a sham injection condition. We recognize that we cannot comment on participants' attention states so we revised the text to speculate on how receiving an injection could potentially change the salience of the injected hand without mentioning attention. "Accordingly, the task performance and gaze behavior changes could be attributable simply to an increased salience of the non-dominant hand because it received an injection rather than reflecting the loss of sensation on that hand." Q1.4 Additionally, I found the following sentence (Page 14, line 335) ambiguous: "From all models, we simplified the fixed effects, random effects, or both to fit the most comprehensive model that still converged." It is unclear what is meant by "simplified." Typically, model selection is guided by balancing goodness-of-fit and parsimony, often using criteria such as AIC or BIC to avoid overfitting. The authors do not report such comparisons, and the inclusion of additional complexity (e.g., Phase and Hand) may result in a model that is unnecessarily complicated given the small sample size. This is especially relevant in light of the three-way interaction the authors found in their results.

Our primary goal is to characterize the effects of anesthesia, which has been modeled by the Hand*Block interactions. In response to a reviewer's comment on our first submission, we added additional random effects to our models to capture subject-specific variations. The inclusion of these random effects resulted in a convergence failure for some tests. In order to achieve model convergence (and interpretable results), we dropped model parameters addressing random effects that were not critical to our primary hypotheses. This strategy is consistent with the approach described in Barr et al., 2013; "Random effects structure for confirmatory hypothesis testing: Keep it maximal". Please note that we could report only the simple models included in our initial submission. In the current revised manuscript, all reported models can be found in our GitHub repository (now shared). "The code/software described in the paper is freely available online at https://github.com/YauLab/NumbPeg." Q1.5 A further concern relates to the factor "Block," whose levels differ across experiments. As stated on Page 13, line 317: "we included block (Experiment 1: baseline vs post-anesthesia; Experiment 2: baseline vs sham vs anesthesia)" This implies that the model is estimating a three-way interaction using a factor "Block" that has a different levels in each experiment: experiment 1 contains two sublevels (baseline vs post-anesthesia) and experiment 2 with 3 sublevels (baseline vs sham vs anesthesia). In such cases, it would be more appropriate to treat Experiment (or "Hand," as currently defined in the manuscript) as a random effect to reflect the hierarchical structure of the data. However, this would lead to a further problematic issue related to the low number of participants in each experiment.

We apologize for confusion. No subject participated in both experiments, so the analyses are performed for each experiment separately. Because Experiments 1 and 2 are analyzed separately, there is no concern that there are different levels of the Block factor in the experiments.

Q1.6 The explanation of "series" and "rounds" is unclear. It is not evident what distinguishes a "series" from a "round," and why the authors chose to model Series using the random effect structure (0 | Subject/Series). This implies that each subject has a unique series, which does not seem to be the case. The pegs were positioned in the same way for each subject and in the same position. If each subject performed the same set of peg movements (i.e., no variability in the items or task structure), a more suitable approach to analyzing learning effects would be to either:

1) Model trial number as a fixed effect to assess learning trends over time (e.g., by slope), or 2) Bin trials into meaningful chunks and include them as a categorical factor to capture non-linear learning trajectories.

As currently implemented, the model structure appears misaligned with the experimental design, potentially undermining the interpretability of the findings.

The learning effect is particularly relevant given the findings in Dempsey-Jones, H., Themistocleous, A. C., Carone, D., Ng, T. W. C., Harrar, V., &Makin, T. R. (2019). Blocking tactile input to one finger using anaesthetic enhances touch perception and learning in other fingers.Journal of Experimental Psychology: General, 148(4), 713-727. https://doi.org/10.1037/xge0000514.

In response to Q1.1, we clarified the structure and terminology of our experimental design (see revised Fig. 1D).

We apologize for the confusion regarding the random effect structure that was wrong. We have corrected the random effect structure reported in the methods to (0 | Subject/Round).

Although our primary questions are not oriented around learning effects, we considered them based on the reviewer's comment. We evaluated whether a metric of task performance (duration of force application) and a metric related to gaze-hand relationships (GISI at peak force) changed as a function of the 20 series completed within each block. For this analysis, our prediction that learning effects - if present - would reflect increased efficiency, such that force duration would decrease across series and GISI would increase.

To quantify potential learning effects on force duration or GISI (dependent variables) under each condition (i.e., "Does force duration with the dominant hand change over the 20 series comprising the anesthesia block?"), we fit the following model:

DV=Block*Hand*Series_Block+(1|Subject/Round) The trend (slope) was calculated with the emtrends function from the emmeans package to identify significant changes of force duration or GISI at peak force as a function of series.

Although we found significant series-related changes in force duration and GISI in some conditions, these effects were inconsistent across experiments and tended to be small. Some trends followed learning effect predictions (e.g., force duration decreases with the anesthetized hand in Experiment 2). Critically, these changes were substantially smaller than changes associated with anesthesia effects. Trends were also observed that were inconsistent with learning. Because learning effects are secondary to our primary questions of interest and given their diminutive size and inconsistency, we have revised the manuscript to include only a brief consideration of learning effects in the Discussion: "Notably, the gaze-hand separation in some actions increased following sham injection compared to baseline, suggesting that participants relied less on their gaze as they gained experience on the task. Indeed, we found a significant Block*Hand interaction in the collect and delivery actions in the placement phase and the collection action in the retrieval phase, which is consistent with learning effects within the blocks (Wolpert &Flanagan, 2010). Motivated by these observations, we performed exploratory analyses to quantify potential learning effects. We tested whether the duration of force application and GISI at peak force changed within each block for each hand separately. Although we found significant linear trends in some conditions, these effects were inconsistent across experiments and tended to be small. Some trends followed learning effect predictions (i.e., shorter force durations and increased GISI over series); however, trends in the opposite direction were also observed. Crucially, any changes in force duration or GISI associated with potential learning effects were substantially smaller than digital anesthesia effects." For the reviewers, we provide the statistical results and visualization of the trends below:

Q1.7 There is no mention of the movement of neighbouring fingers in peg placement. It could be that eye movements were synchronized to the movements of the intact fingers rather than the anesthetized ones.

We thank the reviewer for this thoughtful comment. While we cannot definitively exclude the possibility that participants occasionally directed their gaze toward the annular and little fingers during peg manipulation, several factors suggest this was unlikely. These fingers did not participate in grasping the pegs in any of the trials, as confirmed by continuous visual monitoring by the experimenter. The task consistently elicited a precision grip involving the thumb, index, and middle finger-that is, the anesthetized digits. Moreover, due to the natural hand orientation during the task (held in front of the body), the annular and little fingers were partially occluded by the radial digits. This positioning further reduces the likelihood that these fingers were the primary focus of gaze during object manipulation.

That said, we recognize that without direct quantification of finger-specific gaze allocation or visibility, we cannot fully rule out the influence of neighboring finger movement on visual attention. To address this limitation, we have revised the discussion to explicitly note this potential confound. "Furthermore, we did not track the non-anesthetized fingers and participants may have shifted their gaze to these fingers. We think this is unlikely for multiple reasons. First, due to the natural hand orientation during the task, these fingers were partially occluded by the radial digits. Continuous visual monitoring by the experimenter also confirmed that these fingers did not participant in grasping the pegs in any of the trials. However, without direct quantification of finger-specific gaze allocation or visibility, we cannot rule out the influence of neighboring finger movement." Q1.8 Regarding the peg position, it is possible that eye-hand coordination changes according to the order in which the pegs are picked up and placed. It may be that those who start with the peg closest to the hand start position have a different strategy than those who start with the peg positioned far from the hand start position. Were there any instructions given to the participants regarding the first pegs to pick up, in this case? No instructions were provided to participants regarding the first pegs to pick up. We have added this detail to the revised manuscript: "No instructions were given regarding the order to collect the pegs (other than one at a time) or regarding gaze direction (i.e., the participants were free to find their own strategy)." "Third, the lack of consistent trial structure, particularly with respect to the peg order that participants needed to complete the delivery and collection actions, precluded us from careful analysis into the pattern of gaze behavior based on pegboard position. Future studies incorporating a systematic peg selection order may better assess the impact of visuomotor strategy on eye-hand coordination behavior and motor planning." Q1.9 The description of the procedure and methods is quite confusing. On page 14, line 348, the authors state:"The task was split into two phases: placement or retrieval of pegs. Within each phase, we defined 4 actions using the force signals measured at the collection trays and pegboard: peg-free hand movement, peg collection, peg transport, and peg delivery."Figure 4 is meant to represent these four actions, with each action depicted as a signal ranging from 0 to 100%. However, on page 8, line 196, the authors write:"For each trial, we identified the timing of collection, transport, and delivery events based on the force variations measured on the collection and peg-board trays."This creates confusion. Figure 4 suggests that each of the 4 "actions" unfolds continuously over time, represented as a full signal spanning from 0% to 100% of the movement duration. In contrast, the description on page 8 implies that these actions are discrete events that occur at specific time points along the hand trajectory. Given this, I expected to see clear time-stamped data for each distinct event (peg-free movement, collection, transport, and delivery) rather than continuous signals. The inconsistency between the figure and the textual description makes the methodology unclear and difficult to interpret.

Thank you for pointing this out. We have clarified the relevant section. The data were continuously recorded (as depicted in Fig. 1C) over a round. Using the force transducer signals, we defined the beginning and end of each action (e.g., The beginning and end of the collection action was defined by the onset and offset of force on the collection tray, respectively.) Accordingly, each action unfolds in time. Because the duration of the actions varied, each of these actions were normalized by its duration (0 to 100%) for analysis. We have edited the manuscript to be more explicit that the data were continuously recorded and how the data were subsequently split into non-overlapping actions. We have also edited the figures to show only the discrete actions such that there is less ambiguity: "Data were continuously recorded over each round." "From the continuously recorded data, we segmented the placement and retrieval phases into discrete collection, transport, and delivery actions objectively using the normal force signals measured at the pegboard and collection trays." Minor comments Q1.10 It would have been useful to have the changes tracked by evidencing them in a color other than black. This would have eased the comparison with a previous version of the manuscript.

We submitted a version of the manuscript with changes tracked in highlights in accordance with eNeuro guidelines.

Q1.11 Page 5 line 105: it may be worth repeating that Experiment 1 was related to the non-dominant hand, and Experiment 2 to the dominant hand by stating it within parentheses, for instance: (Experiment 1 - non-dominant hand) This information is provided in the revised manuscript: "Five participants performed the task (Experiment 1) under baseline (no sham or anesthesia) conditions and after digital anesthesia to the non-dominant hand. A separate sample of 6 participants performed the identical task (Experiment 2) under baseline conditions, saline injection to the dominant hand (sham), and digital anesthesia to the dominant hand." Q1.12 Page 12 line 281:" This distinction allowed us to test the hypothesis that gaze-hand position relationships depend on the task difficulty" The task difficulty, however, was not modeled in the mixed model. This is a bit confusing.

We have edited the text to remove allusions to task difficulty: "This distinction allowed us to test the hypothesis that gaze-hand position relationships depend on the action being performed." Reviewer 2 Major Comments Q2.1 I understand the use of trial-level data to allow complex modelling with a limited sample size. However, I do still have remaining concerns about just how small the sample is vs the complexity of the models fitted. Estimates of the between-subjects variability is likely underpowered. Relatedly, the statement in the discussion "Our data revealed high consistency across participants within each experiment" does not seem to have been demonstrated by the results provided. More caution should be taken in the discussion regarding the limits of generalisability based on the small sample.

We acknowledge the reviewer's concerns. Based on comments from Reviewer 1, we performed power analyses through simulations that tested the exact LME specifications in our reported analyses. Given that our primary conclusions center around results from 3 main analyses, we focused our power analyses on these results: 1) Anesthesia alters task performance as measured by the duration of force application, 2) Anesthesia is associated with a reduction in the separation between gaze and hand position when participants engage with the pegs as measured by GISI at peak force application, and 3) Anesthesia is associated with GISI reduction during hand movements with and without pegs. Explicitly, our power analyses determined how likely the null hypothesis can be rejected at different sample sizes given our observed effects size assuming the trial number and variability in our data. For each analysis, we simulated the experiments 1000 times for each sample size comparing a model with the critical interaction term to a model assuming no interaction. In the analyses reported in the main text and supplement, significant effects were reported in 22/24 tests. Power analyses revealed that >99% power is achieved with 4 subjects for 20/22 significant effects we reported. For the remaining 2 reported significant results, >80% power is achieved with 4 subjects. These analyses provide confidence in our main conclusions despite the variability in our small datasets.

We agree with the reviewer that claiming high consistency may be unwarranted so we have removed that language. Additionally, although confidence in our conclusions given our sample is bolstered by the power analysis results, we agree that claims about generalizability should be tempered. We have edited the discussion to reflect these acknowledgements and caveats: "Our data revealed robust effects of anesthesia that replicated over two independent experiments. Although there were subtle individual differences that likely reflected the strategies employed to complete the task, we leveraged this variance in our models which included trial-level data from the experimental and control hands of each subject. Despite confidence in the results of our small-sampled experiments, the generalizability of our conclusions must be evaluated in future studies with larger samples." Q2.2 Additionally, I did not receive access to the "code included as Extended Data" that should have included the final models for each analysis. Could the authors please provide the final models for each? Was it always ensured that the repeated-measures structure of the data was appropriately accounted for? Our analyses always accounted for the repeated-measures structure of our data. We have provided the URL to the GitHub repository where the code and data are uploaded. "The code/software described in the paper is freely available online at https://github.com/YauLab/NumbPeg." Q2.3 I could not locate the statistical analysis for the time taken and force production, that led to the statement "digital anesthesia resulted in significant changes in the time required to complete the actions and the forces produced by the actions" (line 359-360). Could the authors please ensure this analysis is included somewhere - I think the manuscript would benefitting from including this result in the main text, instead of just being included in the extended data.

Statistical analyses related to the force data were removed from the main text to address a reviewer's comment on our initial submission. Consistent with that reviewer, we feel that addressing the force analysis results in the extended data helps to focus the manuscript on new results. We apologize for not including the statistical analyses in the extended data with our previous revision. We now include a summary table of the statistics along with the force data within the extended data. We also provide the raw data and analysis code in the GitHub repository.

Q2.4 Each set of post-hoc tests following a significant interaction should be corrected for multiple comparisons.

We used the emmeans package which determines appropriate application of multiple comparisons correction, defaulting to Tukey's test. "Post hoc tests were calculated using the emmeans package (Lenth, 2017; Searle et al., 1980), which applies multiple comparisons correction (Tukey's test)." Q2.5 Did the authors observe any correlations between GISI changes and time taken or force modulation in the anaesthetised hand? Based on this question, we examined the correlation of the subject-level means between GISI at peak force and peak force and between GISI at peak force and force duration in the anesthetized hand across all actions (coarse collect, precise delivery, precise collect, and coarse delivery) for Experiment 1 and Experiment 2. This yielded 16 total tests. No correlations were determined to be significant. The average correlation in Experiment 1 tests was 0.05 = 0.57. The average correlation in Experiment 2 tests was 0.09 = 0.50.

Minor Comments Q2.6 Were participants specifically instructed to use a precision grip with their Thumb and index/middle, or given that it was strategy-free, would it have been allowed to compensate by using a non-anaesthetised finger, e.g. Thumb with the ring finger? If so, were such compensatory behaviours observed? Participants were only instructed to use a precision grip. We did not observe alternative strategies. We revised the manuscript to clarify the instructions provided to participants: "Participants were instructed to use a precision grip to grasp and place the pegs." Q2.7 Were participants blinded to when they were receiving the sham injection vs the anaesthetic, or were they told the ingredients of each set of injections before they were administered? Although participants were not informed about the nature of the injections they received before each block, they were also not explicitly blinded to the condition (sham vs. anesthetic) given that the effects of the local anesthetic were readily apparent to the individual due to the resulting sensory loss.

Q2.8 Was the 2PD threshold always exceeding 15mm after the anaesthesia (consistent across every single participant)? I would like to see the thresholds visualised as much as possible with the data available, including at baseline vs after the sham injections. Additionally, was the participant's 2PD threshold re-assessed following the final anesthetised block, to assess maintenance of the deafferentation? The 2PD thresholds were re-assessed following the final block. These assessments confirmed that the anesthetic state was maintained throughout the final block.

2PD thresholds always exceeded 15mm after anesthesia. This clearly differs from baseline/sham assessments on the treated hand and assessments on the untreated hand throughout.

Below, we provide the summary statistics for the 2PD thresholds for each experiment. Given the coarseness of the assessments (i.e., 1-mm test resolution below 5 mm) and stable thresholds in all conditions not including anesthesia, the means and variance values do not appear to change. Accordingly, we rely on these measurements only for a qualitative evaluation of sensory capabilities as stated in the text.

Full summary of measurements:

Each digit measured in radial and ulnar half of the volar aspect of the distal phalanx, i.e, 6 measurements per hand for each measured time point.

Experiment 1 Average 2PD (digits 1-3):

Time Point Anesthetized Hand (AH) Other Hand (OH) Before anesthesia 3.6 mm (SD 0.55) 3.8 mm (SD 0.55) After anesthesia >15 mm (all fingers) 3.8 mm (SD 0.55) After last block >15 mm (all fingers) 3.8 mm (SD 0.55) Comment: One subject had 3 mm in all test sites (except for after anesthesia), one had 3 mm in both sites (radial and ulnar) of the thumb of ND hand but 4 mm in all other test sites, and one had 3 mm in both sites of index and long finger of ND hand but 4 mm in all other test sites.

Experiment 2 Average 2PD (digits 1-3):

Time Point Anesthetized Hand (AH) Other Hand (OH) Start 3.83 mm (SD 0.41) 3.83 mm (SD 0.41) After sham 3.83 mm (SD 0.41) 3.83 mm (SD 0.41) After anesthesia >15 mm (all fingers, SD 0) 3.83 mm (SD 0.41) After last block >15 mm (all fingers, SD 0) 3.83 mm (SD 0.41) Comment: One subject had 3 mm in all test sites, the others 4 mm (except for after anesthesia) Q2.9 Once no force is being applied to the force transducers, how long does it take the normal force to return to baseline levels (the latency)? The latency to return to baseline levels is 22.8 = 0.03ms.

Q2.10 Were there any issues with the hand going out of the tracking frame, and if so, how was this accounted for? There were no instances of the hand moving out of the tracking frame during the experiment. The setup was optimized to ensure that the entire workspace for peg placement and retrieve movements remained well within the calibrated tracking volume. We have now clarified this point in the revised methods section. "The device for tracking hand motion (Leap Motion controller, Mountain View, CA, USA; sampling rate: 120 Hz) was mounted centered 40-cm above the workspace (Fig. 1B). The setup was optimized to ensure that the entire workspace for peg placement and retrieve movements remained well within the calibrated tracking volume." Q2.11 The paragraph beginning on line 367 discuss the temporal offset metrics. The paragraph includes results from the "test series" and "retrieval phase" , that appear to be separate time periods within the trial. However, series is earlier defined as the 8 placement and 8 retrieval trials. It is therefore not clear what the difference is in the test series vs the retrieval phase is. Does the test series include everything, or only the placement? We apologize for this confusion. We incorrectly described the analyses as one over series rather than the placement and retrieval phases. We have revised the text to correct this error for Experiment 1 and 2 results: "We first sought to determine how the loss of sensory feedback on the hand modulated eye-hand coordination at the level of series which comprised placement and retrieval phases. Given the cyclical nature of the actions within a phase (sequential engagement of 8 pegs), we reasoned that the temporal offset computed through cross-correlation analysis would provide a metric capturing the relationship between the gaze and hand positions within a phase. We compared the temporal offset metrics computed from each phase completed before (B1) and after digital anesthesia (B2) to determine the general modulatory effects associated with the loss of sensation. Without anesthesia, gaze position routinely preceded that of the hand in the placement phase (158.7 {plus minus} 31.8 ms), consistent with a guidance role for vision. With anesthesia, the offset between the gaze and hand was substantially smaller (73.3 {plus minus} 31.8 ms). A model accounted for these patterns with a significant Block*Hand interaction (-21 = 8.0, p = 0.005). Post hoc tests revealed that the change in offset for the treated hand differed significantly from the change in offset for the untreated hand (∆∆Offset = 58 {plus minus} 21.2 ms, t788 = 2.7, p = 0.006). Notably, anesthesia did not similarly modulate the gaze-hand relationship in the retrieval phase (∆∆Offset = 26.7 {plus minus} 21.2 ms, t788 = 1.3, p = 0.2). The absence of an effect in the retrieval phase may be related to the different gaze demands compared to the placement phase. To address this possibility, we next characterized gaze-hand relationships during each action." "Just as we did for Experiment 1, we first assessed whether anesthesia modulated gaze-hand relationships at the temporal scale of phases. Consistent with Experiment 1 results, smaller offsets were observed for the anesthetized hand compared to the other conditions. A model captured this effect with a significant Block*Hand interaction (-22 = 25.4, p = 10-6). Post hoc tests revealed that the offset change for the treated hand differed significantly from the offset change for the untreated hand (∆∆Offset = 100.0 {plus minus} 21.6 ms, t = 4.6, p < 0.0001). In contrast, anesthesia did not significantly modulate gaze-hand relationships during the retrieval phase (∆∆Offset = 31.67 {plus minus} 21.6 ms, t = 1.5, p = 0.31). These collective results are consistent with the offset results in Experiment 1." Q2.12 The interaction on line 525 does not have any follow-up tests to confirm direction? We have performed a post hoc test for the Block*Hand interaction during coarse delivery in Experiment 1. The post hoc test confirmed that the direction of ∆∆GISI throughout coarse delivery was consistent with that during Peak Force with GISI being lower for the treated hand in B2. "Accordingly, a model revealed a significant Block*Hand interaction (-21 = 506. 9, p < 10-16), but the Block*Hand*Progress interaction (-24 = 3. 5, p = 0.48) failed to achieve significance. Post hoc test on the Block*Hand interaction confirmed that ∆GISI for the anesthetized hand was significantly lower compared to the non-anesthetized hand throughout coarse delivery (∆∆GISI = 43.7 {plus minus} 1.9 mm, z = 22.5, p < 0.0001)." Q2.13 In Figures 2-5, a legend or label for the blue and red bars for the Peak Force would help readability We have included labels within the figures for additional clarity.

Q2.14 In the Extended Data Figures, the legend could be improved for support the reader by stating which colours correspond to which condition (e.g. blue for control, red for anesthetised hand), rather than just 'time 1', 'time 2'.

We have expanded the legend in the Extended Data Figures to include labels for the conditions.
