# Supplementary figures and images for: Acute Loss of Tactile Input Leads to General Compensatory Changes in Eye–Hand Coordination during Object Manipulation
Source: eNeuro. 2025 Sep 25;12(9):ENEURO.0487-23.2025. doi: 10.1523/ENEURO.0487-23.2025 (PMC12494028; doi:10.1523/ENEURO.0487-23.2025)

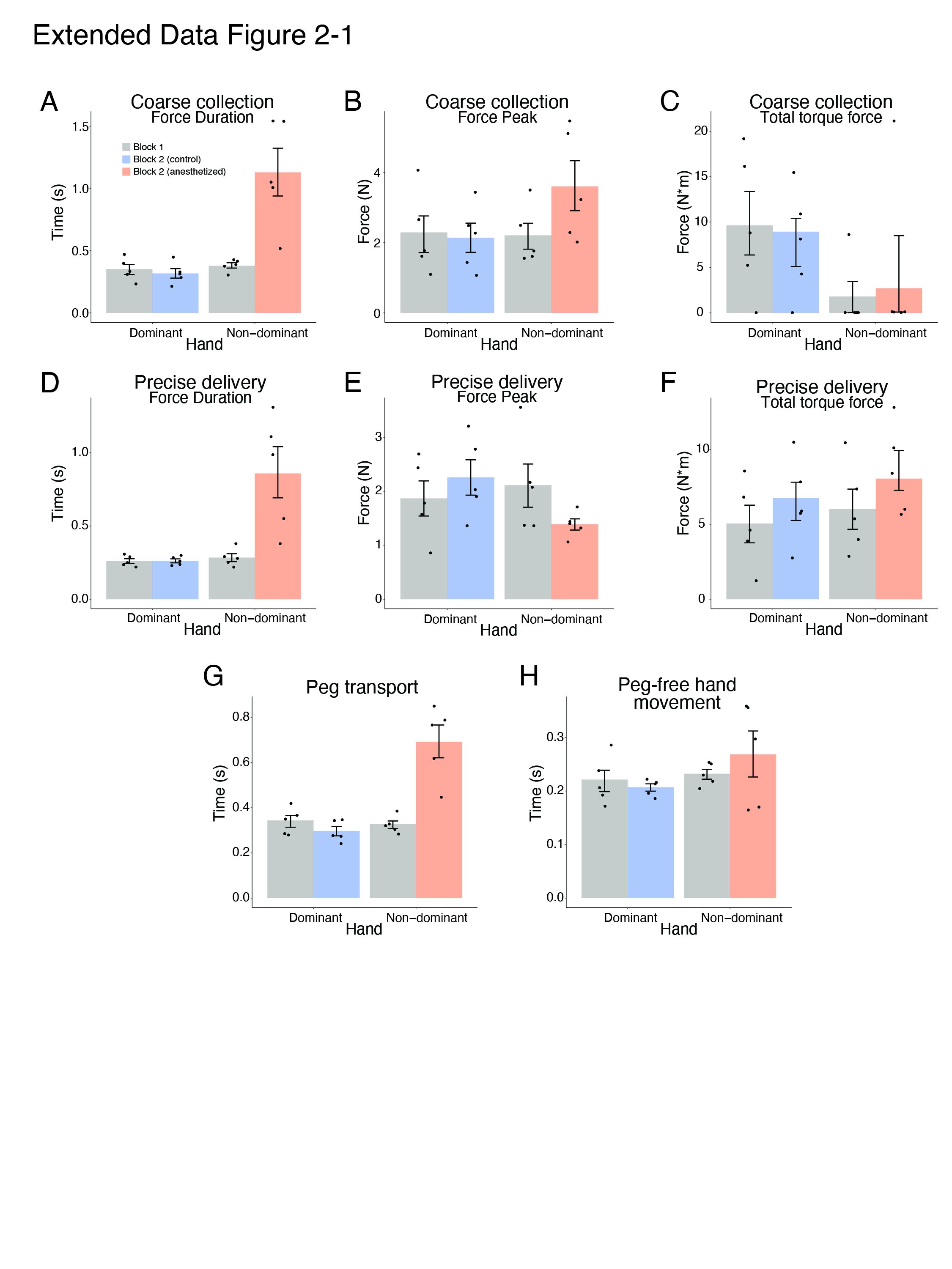

Supplement: Figure 2-1 — Force results for Experiment 1 during the placement phase. Bar graphs of force data during the placement phase, i.e., peg pick-up from tray (coarse collection) and delivery to peg-holes (precise delivery) of experiment 1. (A-C) From coarse collection, bar graphs across all participants and conditions of duration of force application in the collection tray, peak normal force produced in the collection tray, and total torque force in the collection tray. (D-F) From precise delivery, bar graphs across all participants and conditions of duration of force application in the peg-hole, peak normal force produced in the peg-hole, and total torque force in the peg-hole. (G-H) Bar graphs depicting, for all trials sorted by condition, time elapsed between peg transport (completion of peg collection and initiating peg delivery for all trials sorted by condition), and peg-free hand movement (completion of peg delivery and initiating collection of the next peg). Bar graphs represent the mean with individual data points representing individual subjects. Error bar represents s.e.m. Download Figure 2-1, TIF file. [file eneuro-12-ENEURO.0487-23.2025-s002.tif]

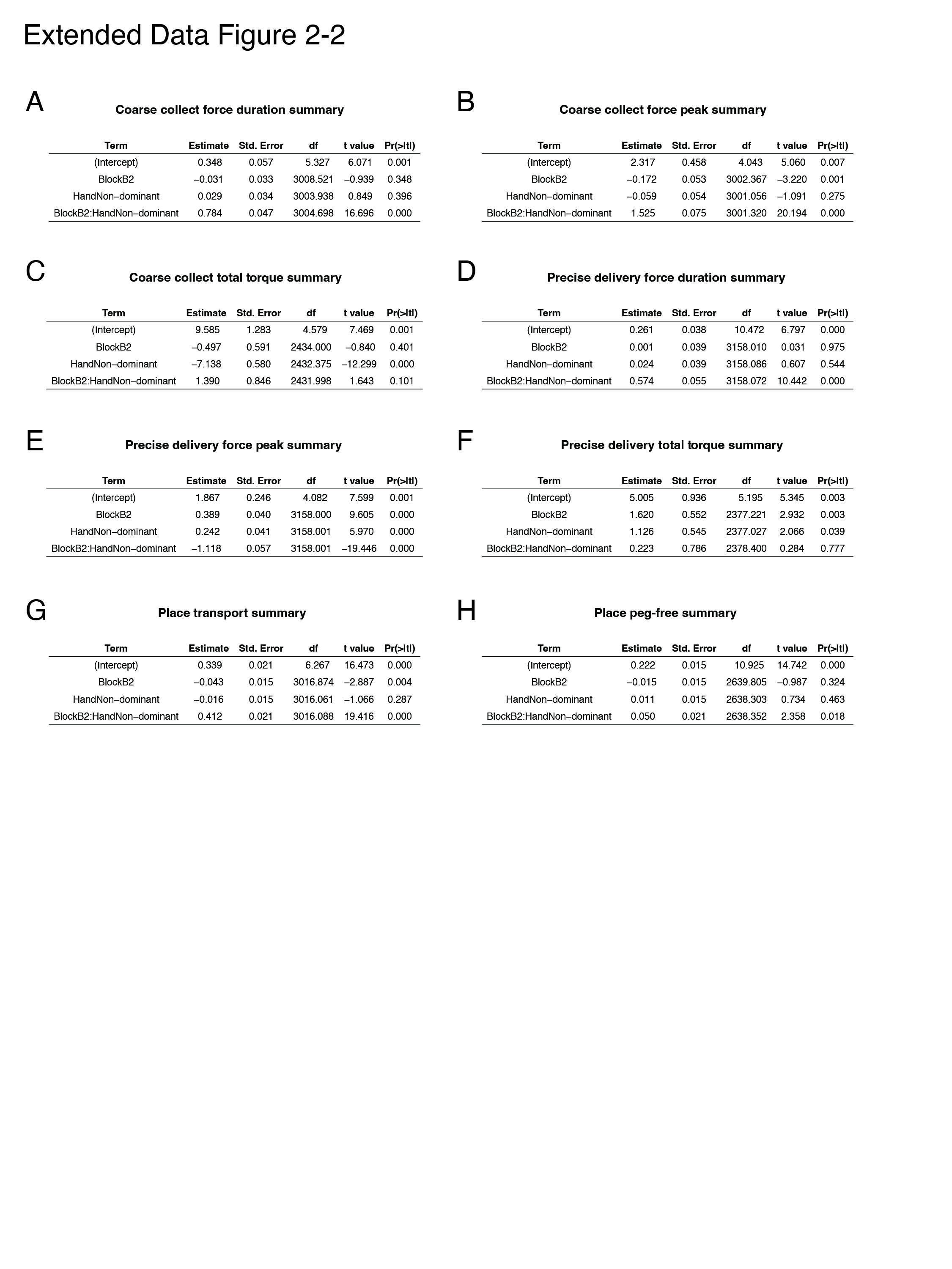

Supplement: Figure 2-2 — Statistical summary of the GLM of the force data for Experiment 1 during the placement phase. (A-C) Statistical results table for force data related to coarse collection during the placement phase. Refer to (A-C) in Extended Data Figure 2-1 for visualized data. (D-F) Statistical results table for force data related to precise delivery during the placement phase. Refer to (D-F) in Extended Data Figure 2-1 for visualized data. (G-H) Statistical results table for transport and peg-free hand movement durations during the placement phase. Refer to (G-H) in Extended Data Figure 2-1 for visualized data. Download Figure 2-2, TIF file. [file eneuro-12-ENEURO.0487-23.2025-s003.tif]

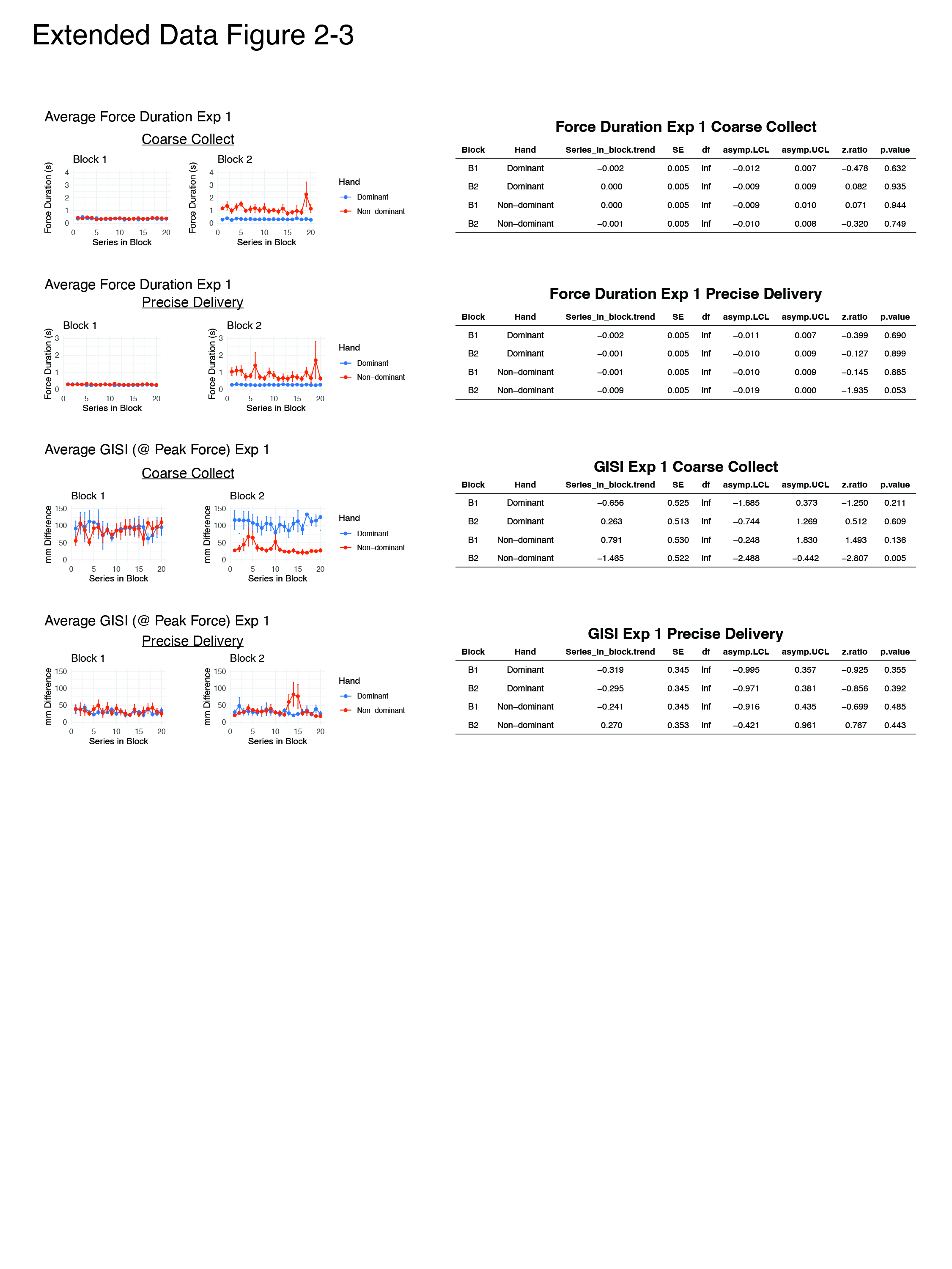

Supplement: Figure 2-3 — Evaluation of potential learning effects on force duration and GISI at peak force during the placement phase in Experiment 1. Traces indicate average force duration and GISI at peak force as a function of series with the anesthetized (red) and unanesthetized (blue) hands during each action. Changes over series were evaluated separately for Block 1 (baseline) and Block 2 (anesthesia). Tables indicate significant and non-significant slopes from linear model fits. For this analysis, we predicted that learning effects – if present – would reflect increased efficiency in task performance, such that force duration would decrease across series and GISI would increase. Significant linear trends were variable, tended to be small, and were inconsistent with learning. Download Figure 2-3, TIF file. [file eneuro-12-ENEURO.0487-23.2025-s004.tif]

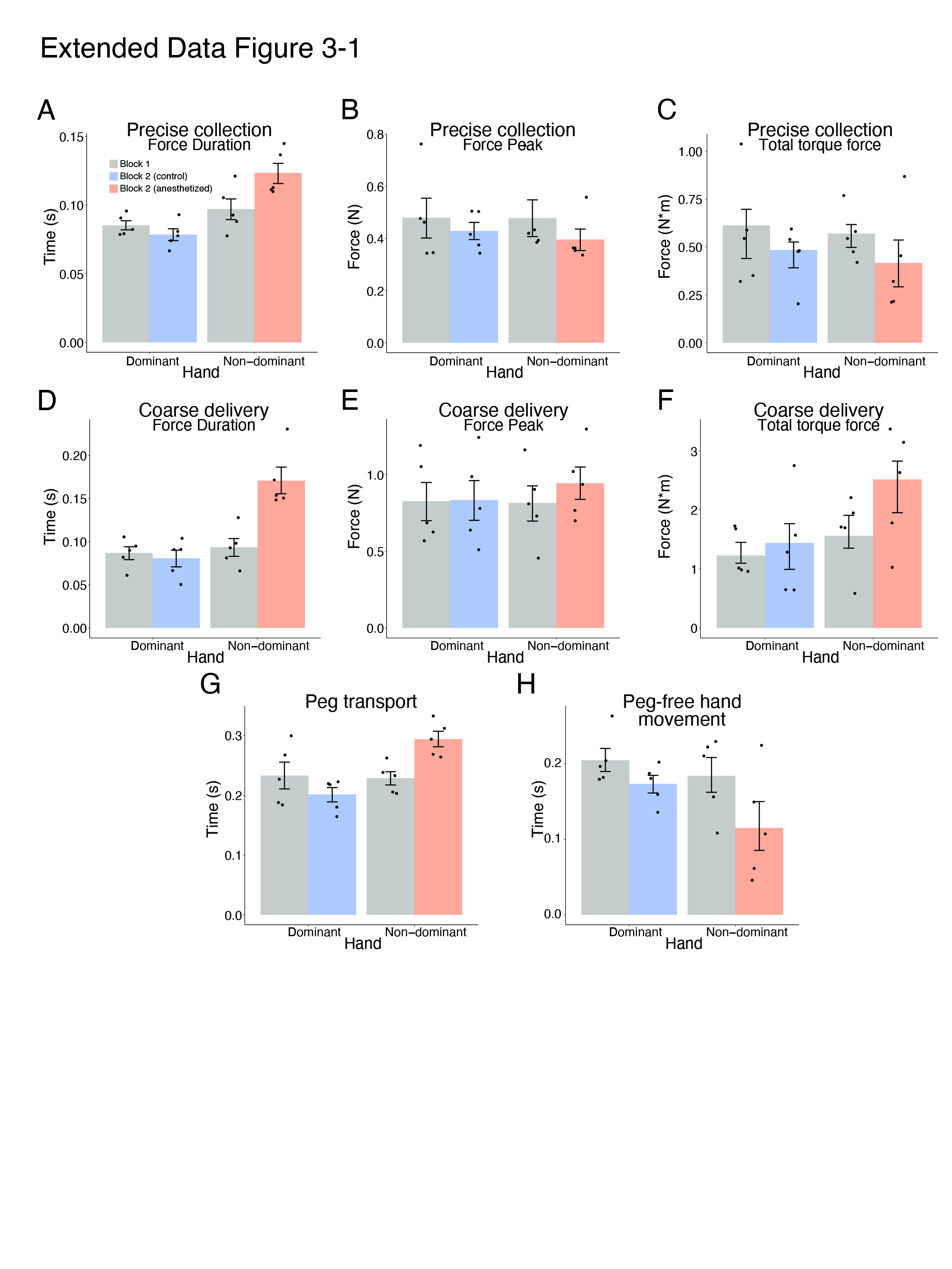

Supplement: Figure 3-1 — Force results for Experiment 1 during the retrieval phase. Bar graphs of force data during the retrieval phase, i.e., peg pick-up from tray (coarse collection) and delivery to peg-holes (precise delivery) of experiment 1. (A-C) From coarse collection, bar graphs across all participants and conditions of duration of force application in the collection tray, peak normal force produced in the collection tray, and total torque force in the collection tray. (D-F) From precise delivery, bar graphs across all participants and conditions of duration of force application in the peg-hole, peak normal force produced in the peg-hole, and total torque force in the peg-hole. (G-H) Bar graphs depicting, for all trials sorted by condition, time elapsed between peg transport (completion of peg collection and initiating peg delivery for all trials sorted by condition), and peg-free hand movement (completion of peg delivery and initiating collection of the next peg). Bar graphs represent the mean with individual data points representing individual subjects. Error bar represents s.e.m. Download Figure 3-1, TIF file. [file eneuro-12-ENEURO.0487-23.2025-s005.tif]

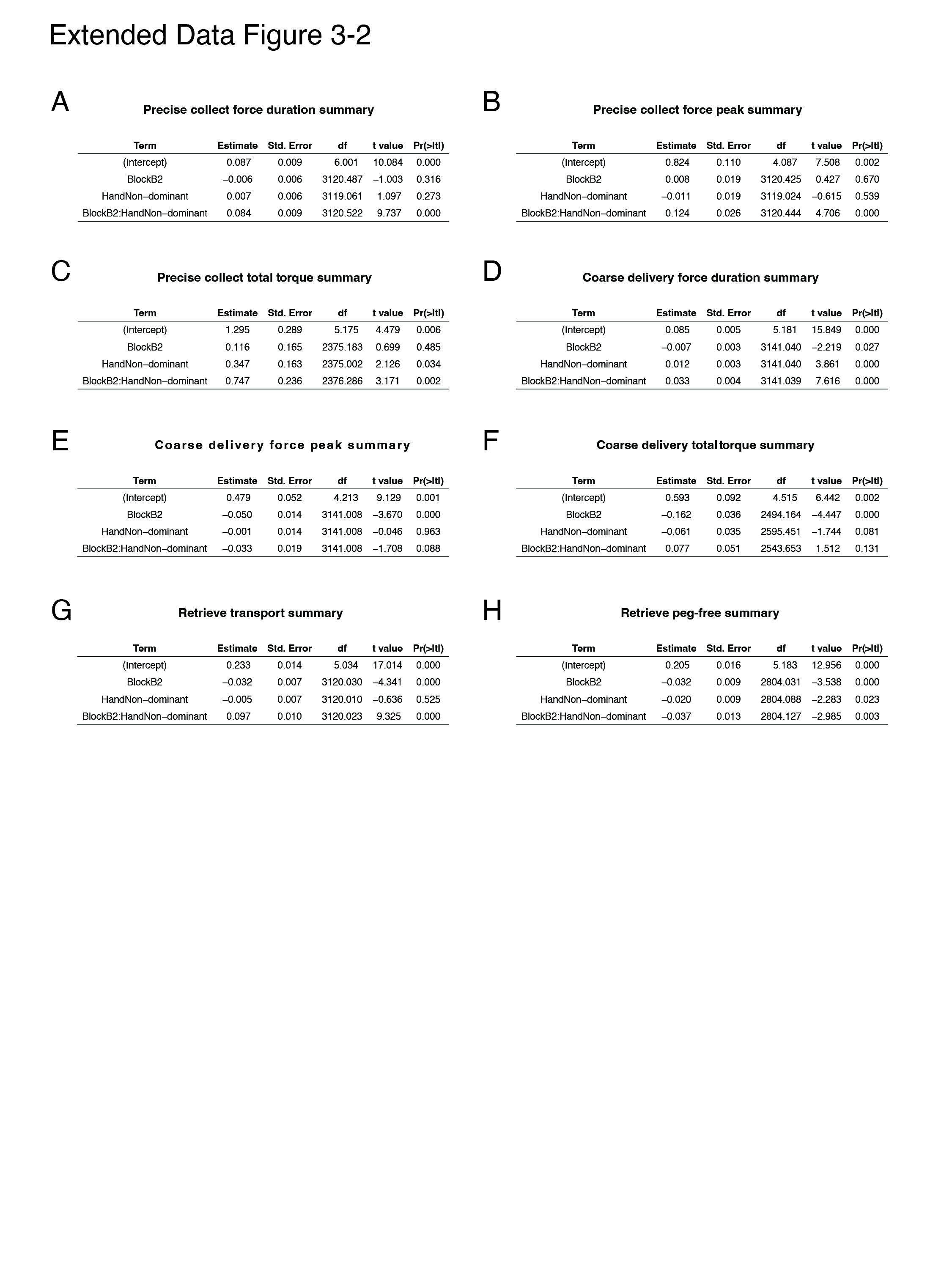

Supplement: Figure 3-2 — Statistical summary of the GLM of the force data for Experiment 1 during the retrieval phase. (A-C) Statistical results table for force data related to precise collection during the retrieval phase. Refer to (A-C) in Extended Data Figure 3-1 for visualized data. (D-F) Statistical results table for force data related to coarse delivery during the retrieval phase. Refer to (D-F) in Extended Data Figure 3-1 for visualized data. (G-H) Statistical results table for transport and peg-free hand movement durations during the retrieval phase. Refer to (G-H) in Extended Data Figure 3-1 for visualized data. Download Figure 3-2, TIF file. [file eneuro-12-ENEURO.0487-23.2025-s006.tif]

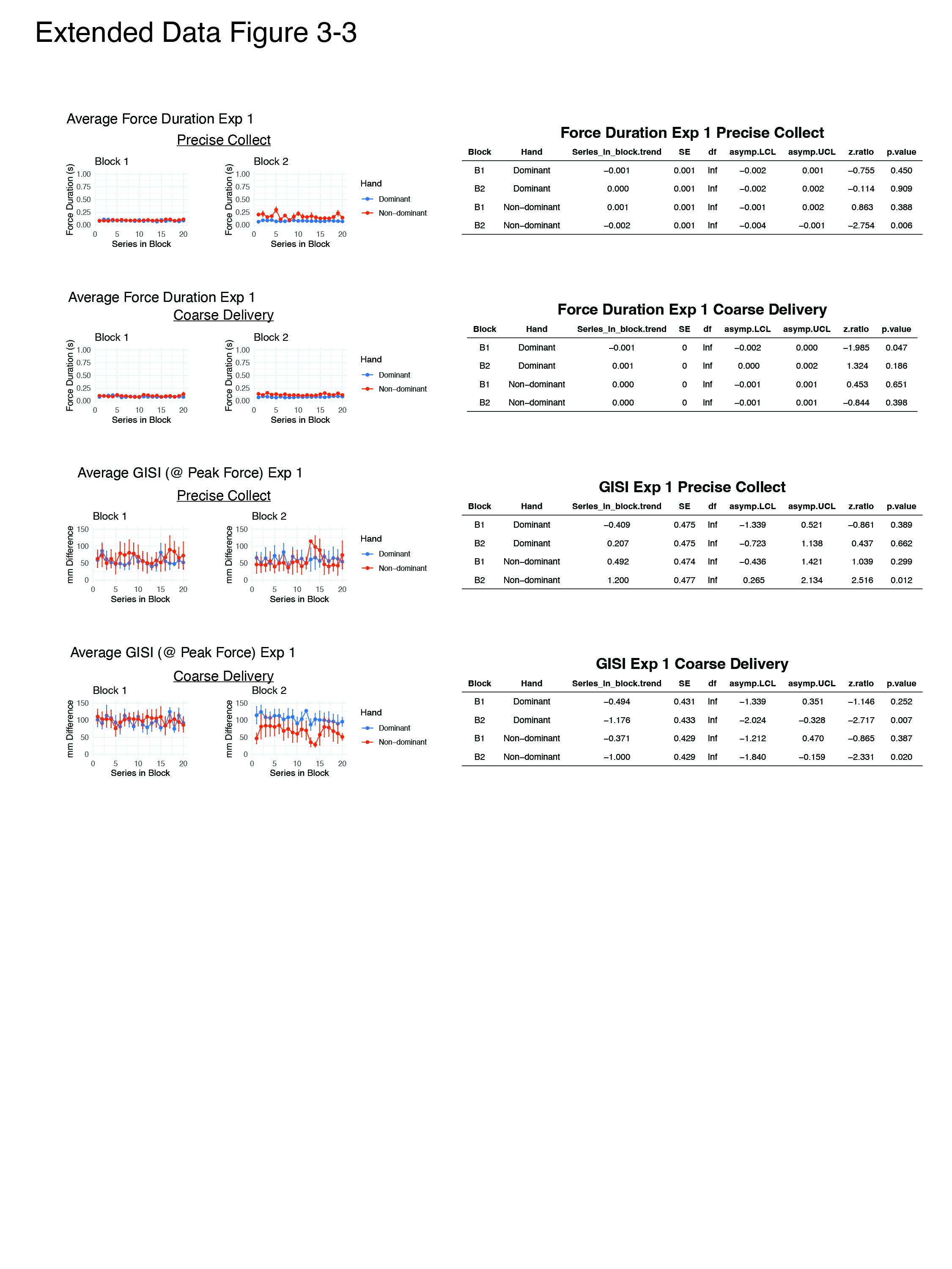

Supplement: Figure 3-3 — Evaluation of potential learning effects on force duration and GISI at peak force during the retrieval phase in Experiment 1. Traces indicate average force duration and GISI at peak force as a function of series with the anesthetized (red) and unanesthetized (blue) hands during each action. Changes over series were evaluated separately for Block 1 (baseline) and Block 2 (anesthesia). Tables indicate significant and non-significant slopes from linear model fits. Download Figure 3-3, TIF file. [file eneuro-12-ENEURO.0487-23.2025-s007.tif]

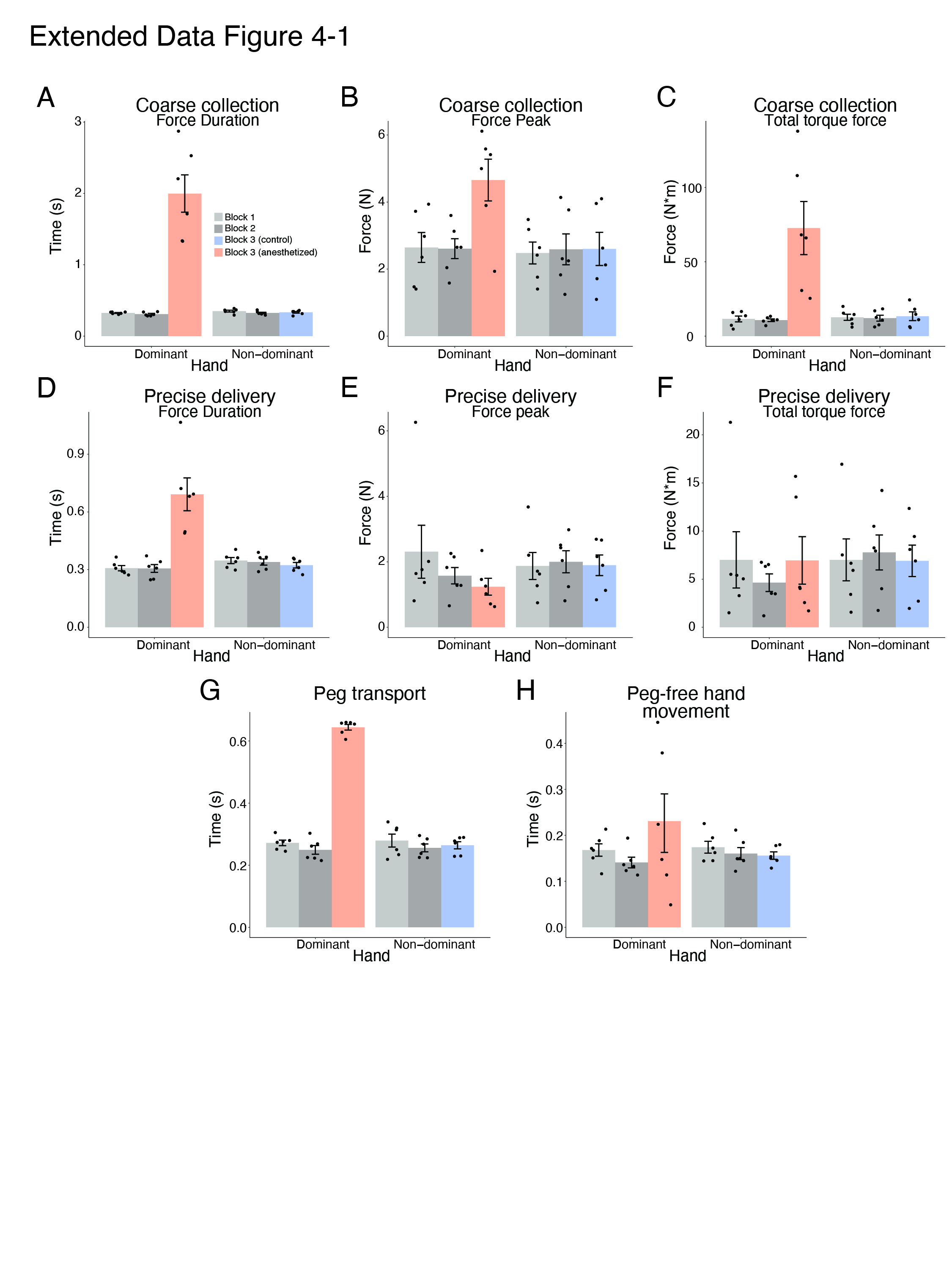

Supplement: Figure 4-1 — Force results for Experiment 2 during the placement phase. Bar graphs of force data during the placement phase, i.e., peg pick-up from tray (coarse collection) and delivery to peg-holes (precise delivery) of experiment 2. (A-C) From coarse collection, bar graphs across all participants and conditions of duration of force application in the collection tray, peak normal force produced in the collection tray, and total torque force in the collection tray. (D-F) From precise delivery, bar graphs across all participants and conditions of duration of force application in the peg-hole, peak normal force produced in the peg-hole, and total torque force in the peg-hole. (G-H) Bar graphs depicting, for all trials sorted by condition, time elapsed between peg transport (completion of peg collection and initiating peg delivery for all trials sorted by condition), and peg-free hand movement (completion of peg delivery and initiating collection of the next peg). Bar graphs represent the mean with individual data points representing individual subjects. Error bar represents s.e.m. Download Figure 4-1, TIF file. [file eneuro-12-ENEURO.0487-23.2025-s008.tif]

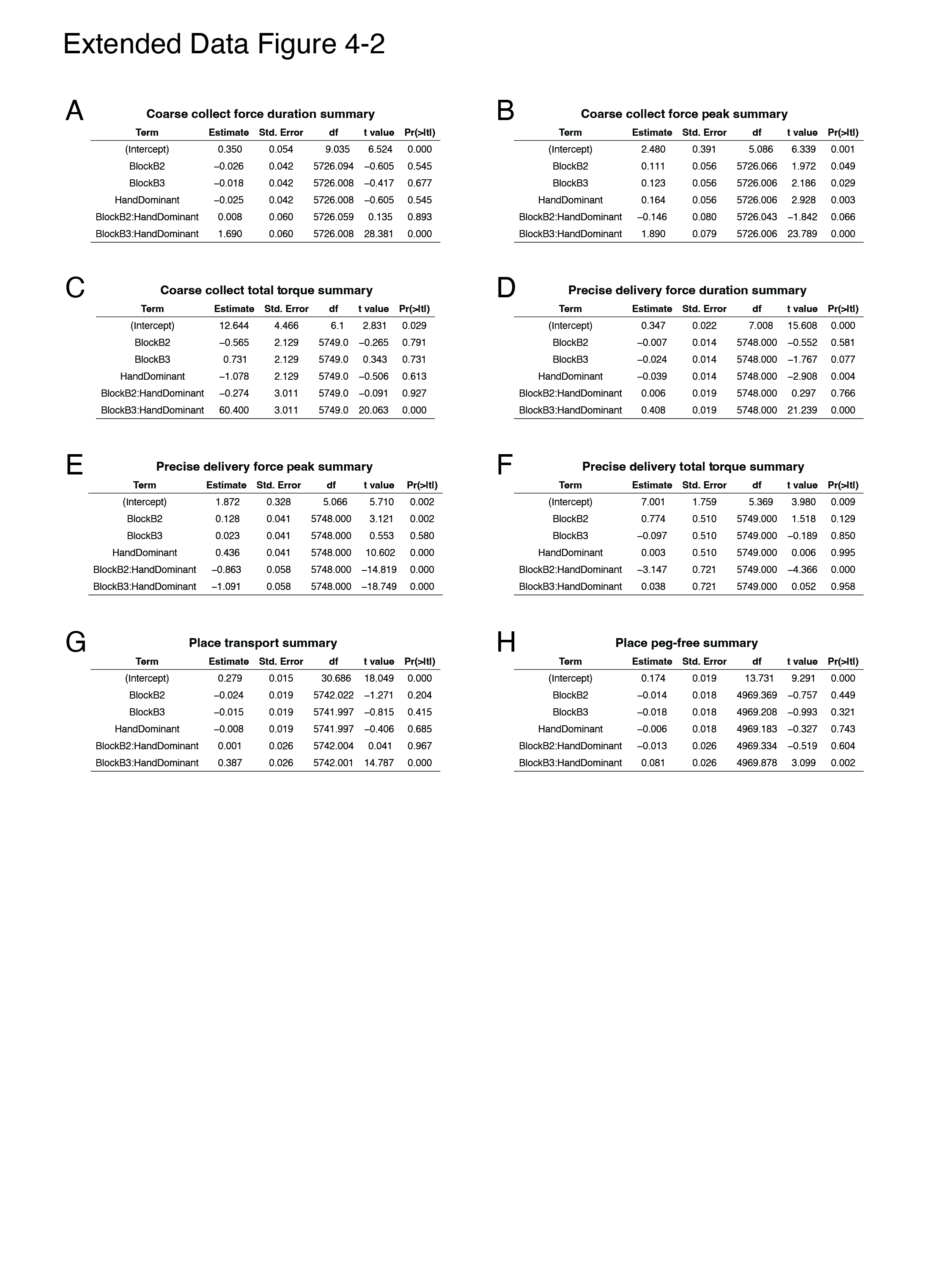

Supplement: Figure 4-2 — Statistical summary of the GLM of the force data for Experiment 2 during the placement phase. (A-C) Statistical results table for force data related to coarse collection during the placement phase. Refer to (A-C) in Extended Data Figure 4-1 for visualized data. (D-F) Statistical results table for force data related to precise delivery during the placement phase. Refer to (D-F) in Extended Data Figure 4-1 for visualized data. (G-H) Statistical results table for transport and peg-free hand movement durations during the placement phase. Refer to (G-H) in Extended Data Figure 4-1 for visualized data. Download Figure 4-2, TIF file. [file eneuro-12-ENEURO.0487-23.2025-s009.tif]

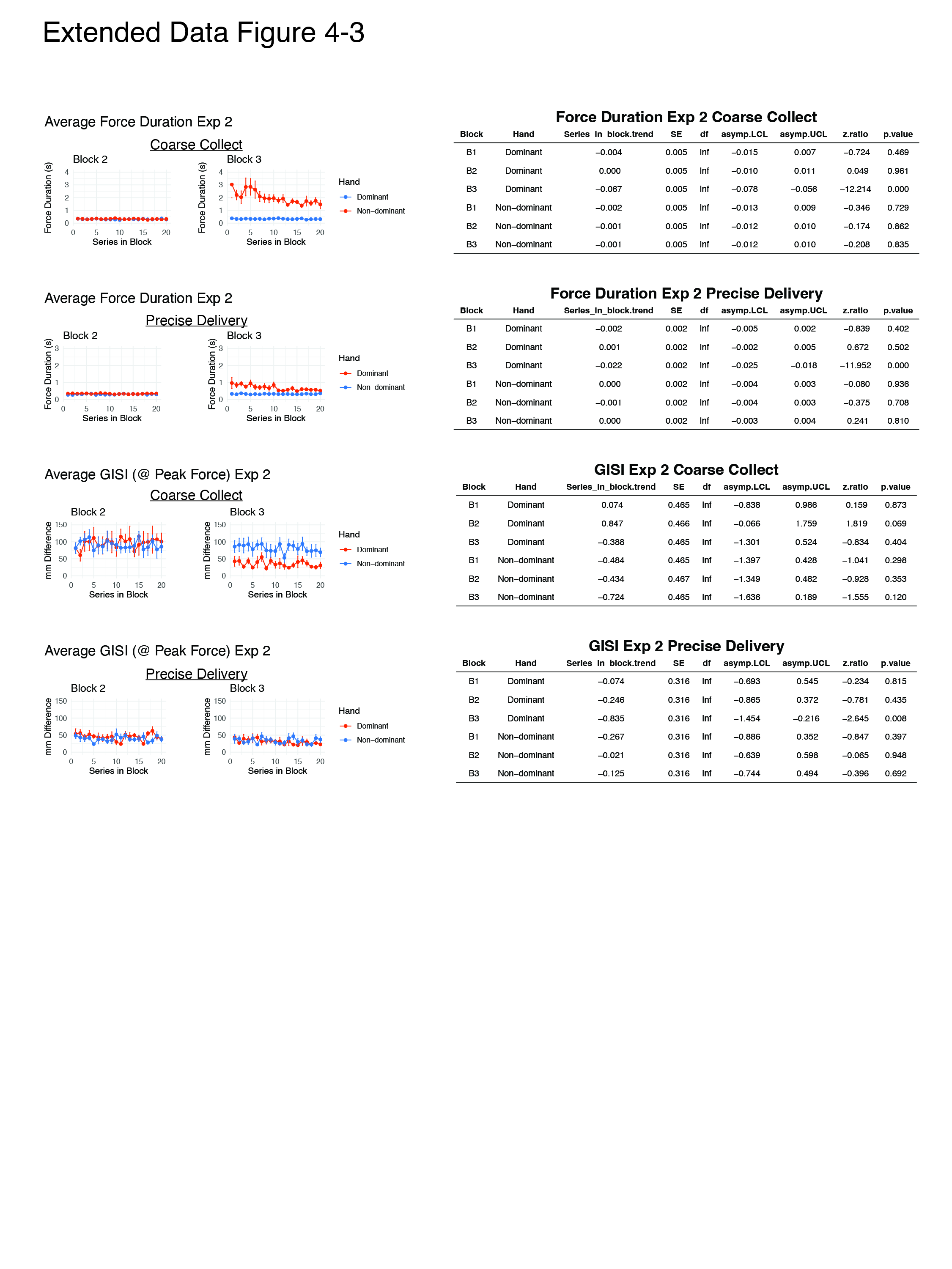

Supplement: Figure 4-3 — Evaluation of potential learning effects on force duration and GISI at peak force during the placement phase in Experiment 2. Traces indicate average force duration and GISI at peak force as a function of series with the anesthetized (red) and unanesthetized (blue) hands during each action for Block 2 (sham) and Block 3 (anesthesia). Tables indicate significant and non-significant slopes from linear model fits from all blocks (B1, B2, B3). Download Figure 4-3, TIF file. [file eneuro-12-ENEURO.0487-23.2025-s010.tif]

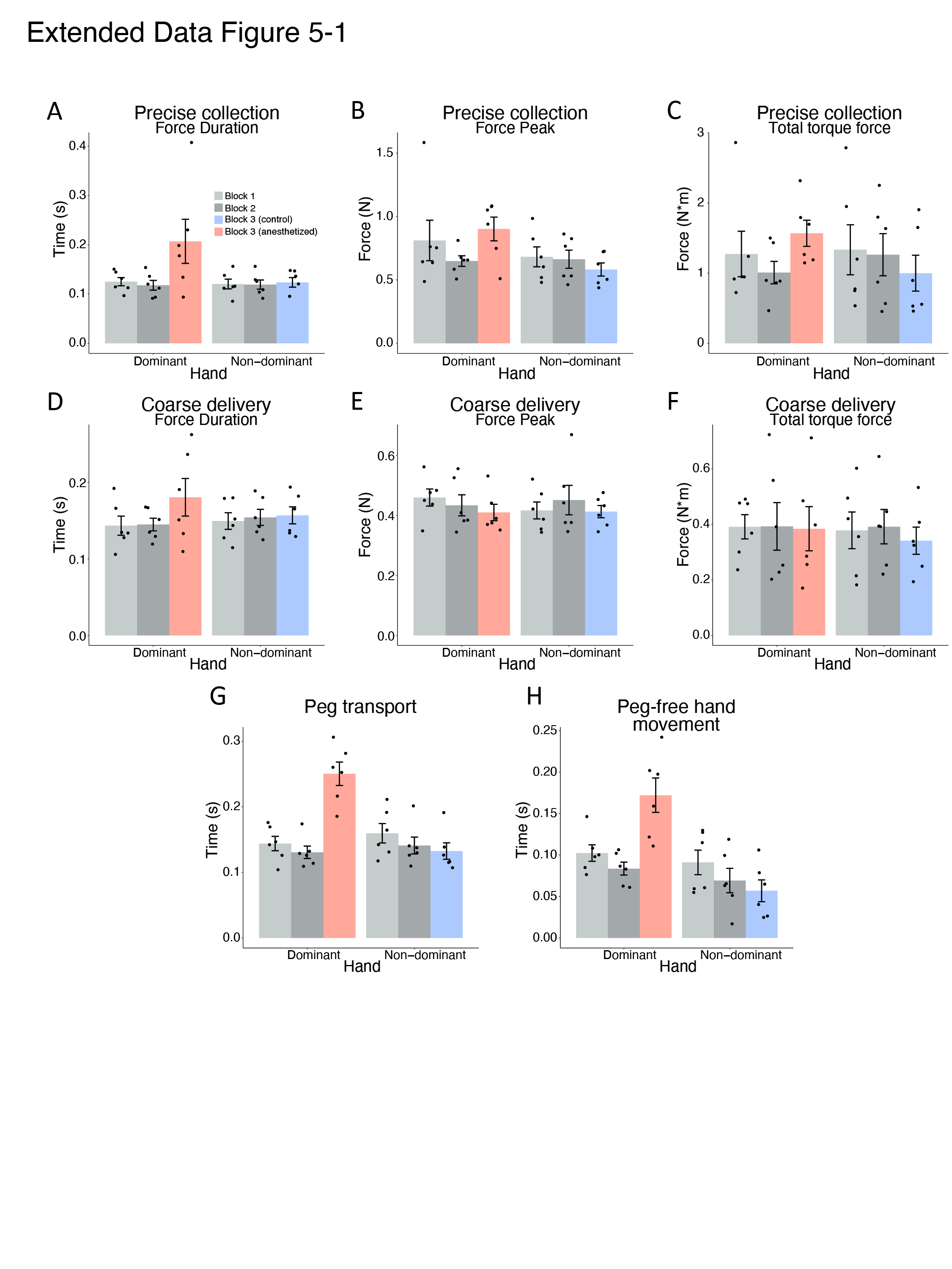

Supplement: Figure 5-1 — Force results for Experiment 2 during the retrieval phase. Bar graphs of force data during the retrieval phase, i.e., peg pick-up from tray (coarse collection) and delivery to peg-holes (precise delivery) of experiment 2. (A-C) From coarse collection, bar graphs across all participants and conditions of duration of force application in the collection tray, peak normal force produced in the collection tray, and total torque force in the collection tray. (D-F) From precise delivery, bar graphs across all participants and conditions of duration of force application in the peg-hole, peak normal force produced in the peg-hole, and total torque force in the peg-hole. (G-H) Bar graphs depicting, for all trials sorted by condition, time elapsed between peg transport (completion of peg collection and initiating peg delivery for all trials sorted by condition), and peg-free hand movement (completion of peg delivery and initiating collection of the next peg). Bar graphs represent the mean with individual data points representing individual subjects. Error bar represents s.e.m. Download Figure 5-1, TIF file. [file eneuro-12-ENEURO.0487-23.2025-s011.tif]

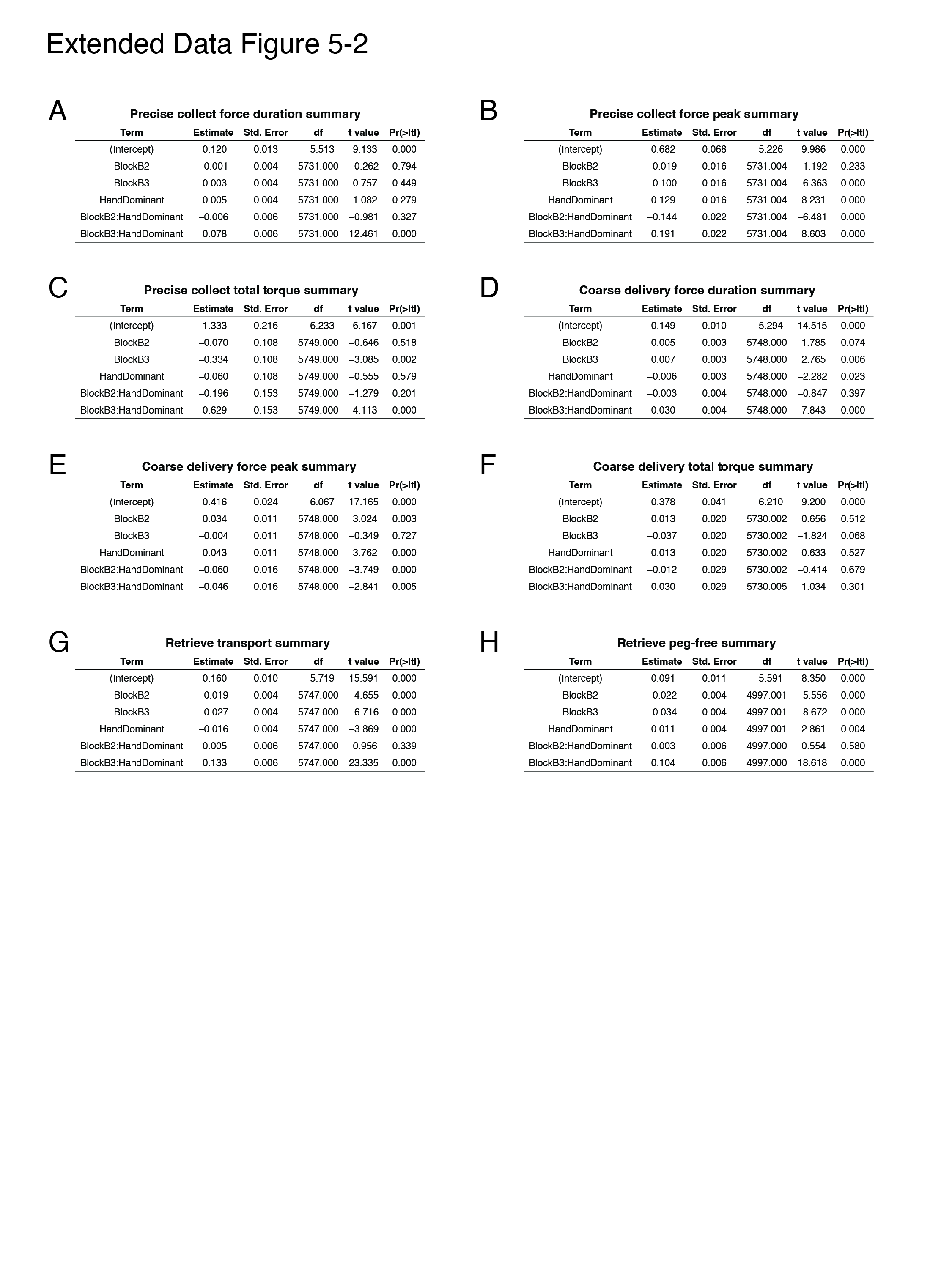

Supplement: Figure 5-2 — Statistical summary of the GLM of the force data for Experiment 2 during the retrieval phase. (A-C) Statistical results table for force data related to precise collection during the retrieval phase. Refer to (A-C) in Extended Data Figure 5-1 for visualized data. (D-F) Statistical results table for force data related to coarse delivery during the retrieval phase. Refer to (D-F) in Extended Data Figure 5-1 for visualized data. (G-H) Statistical results table for transport and peg-free hand movement durations during the retrieval phase. Refer to (G-H) in Extended Data Figure 5-1 for visualized data. Download Figure 5-2, TIF file. [file eneuro-12-ENEURO.0487-23.2025-s012.tif]

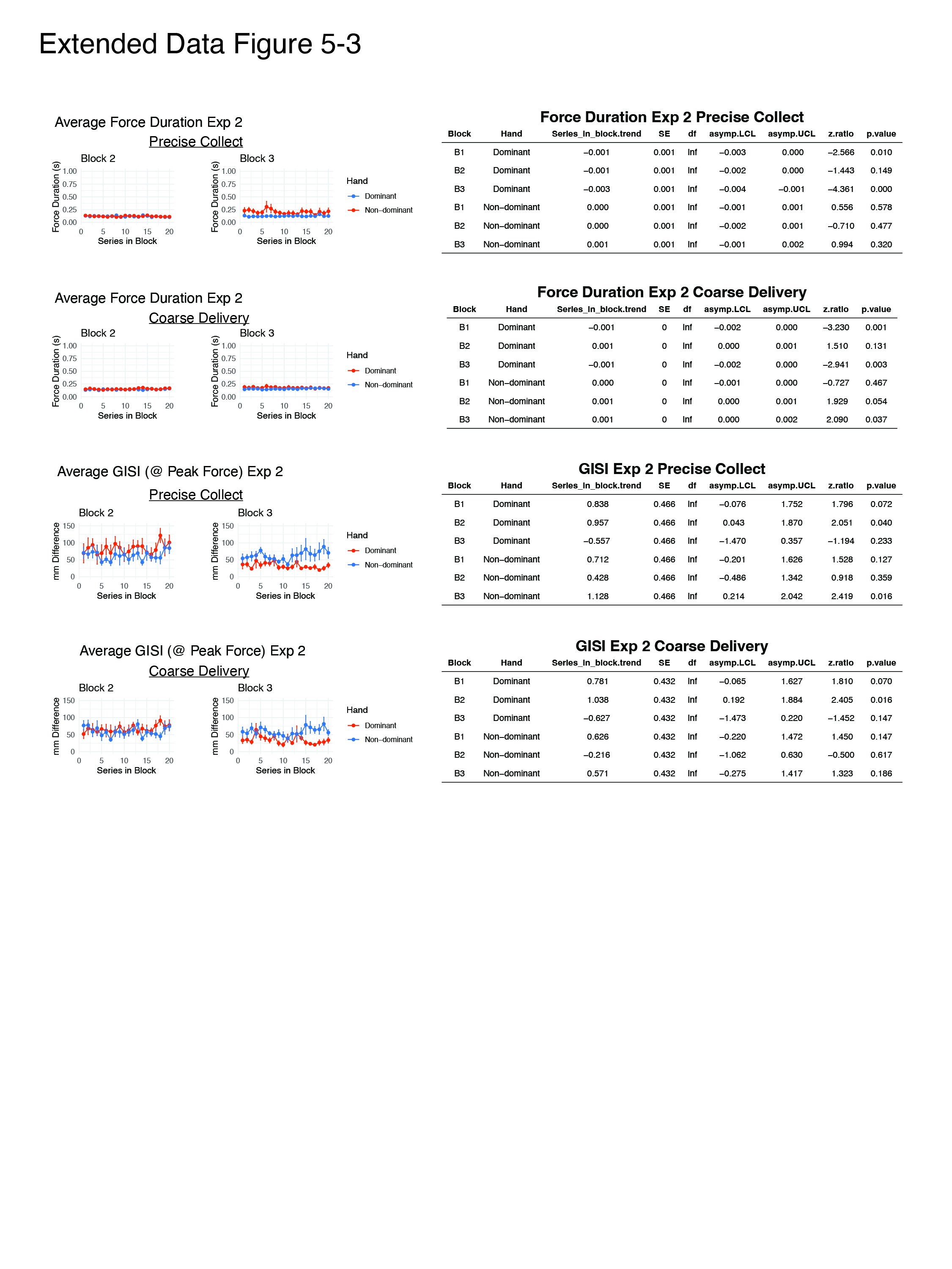

Supplement: Figure 5-3 — Evaluation of potential learning effects on force duration and GISI at peak force during the retrieval phase in Experiment 2. Traces indicate average force duration and GISI at peak force as a function of series with the anesthetized (red) and unanesthetized (blue) hands during each action for Block 2 (sham) and Block 3 (anesthesia). Tables indicate significant and non-significant slopes from linear model fits from all blocks (B1, B2, B3). Download Figure 5-3, TIF file. [file eneuro-12-ENEURO.0487-23.2025-s013.tif]
